# New Trends in Pharmacological Treatments for Osteoarthritis

**DOI:** 10.3389/fphar.2021.645842

**Published:** 2021-04-15

**Authors:** Xiaoyan Cai, Shiwen Yuan, Yanting Zeng, Cuicui Wang, Na Yu, Changhai Ding

**Affiliations:** ^1^ Department of Rheumatology, Guangzhou First People’s Hospital, School of Medicine, South China University of Technology, Guangzhou, China; ^2^ Clinical Research Centre, Zhujiang Hospital, Southern Medical University, Guangzhou, China; ^3^ Menzies Institute for Medical Research, University of Tasmania, Hobart, TAS, Australia

**Keywords:** osteoarthritis, novel therapeutics, DMOADs, therapy selection, clinical prospect

## Abstract

Osteoarthritis (OA) is the leading cause of function loss and disability among the elderly, with significant burden on the individual and society. It is a severe disease for its high disability rates, morbidity, costs, and increased mortality. Multifactorial etiologies contribute to the occurrence and development of OA. The heterogeneous condition poses a challenge for the development of effective treatment for OA; however, emerging treatments are promising to bring benefits for OA management in the future. This narrative review will discuss recent developments of agents for the treatment of OA, including potential disease-modifying osteoarthritis drugs (DMOADs) and novel therapeutics for pain relief. This review will focus more on drugs that have been in clinical trials, as well as attractive drugs with potential applications in preclinical research. In the past few years, it has been realized that a complex interaction of multifactorial mechanisms is involved in the pathophysiology of OA. The authors believe there is no miracle therapeutic strategy fitting for all patients. OA phenotyping would be helpful for therapy selection. A variety of potential therapeutics targeting inflammation mechanisms, cellular senescence, cartilage metabolism, subchondral bone remodeling, and the peripheral nociceptive pathways are expected to reshape the landscape of OA treatment over the next few years. Precise randomized controlled trials (RCTs) are expected to identify the safety and efficacy of novel therapies targeting specific mechanisms in OA patients with specific phenotypes.

## Introduction

Osteoarthritis (OA) can be viewed as the structural and functional failure of the synovial joint organ (Loeser et al., 2012). All tissues of the joint can be involved, including articular cartilage, subchondral bone, and synovium (Felson, 2006). OA is the leading cause of function loss and disability among elderly, which makes these patients suffer from chronic pain (Hunter and Bierma-Zeinstra, 2019). Traditionally the management of OA has been constrained to symptom relieving (Arden et al., 2020); the non-steroidal anti-inflammatory drugs (NSAIDs) or analgesics are most commonly applied to OA for relieving pain, however, their side-effects often restrict their use (Bally et al., 2017; da Costa et al., 2017; Fuggle et al., 2019; Leopoldino et al., 2019). In recent years, there has been substantial progress made in understanding the pathogenesis of OA.

OA is a very complicated pathophysiologic process and is a result of interacting action of multiple mechanisms. Mechanical overload, genetic alterations, sex hormone deficiency, aging, metabolic imbalance and low-grade chronic inflammation all may contribute to the imbalance between catabolism and anabolism of joint tissues, and lead to eventual joint damage in OA. The etiological heterogeneity causes a great difficulty on the development of an effective treatment for OA. The development of OA is a very complicated pathophysiologic process and is a result of interaction of multiple mechanisms. Mechanical overload, genetic alterations, sex hormone deficiency, aging, metabolic imbalance and low-grade chronic inflammation all may contribute to the imbalance between catabolism and anabolism of joint tissues and lead to eventual joint damage in OA (Chen D. et al., 2017; Oo et al., 2018). The etiological heterogeneity causes a great difficulty on the development of an effective treatment for OA. Epidemiological data support significant associations between structural changes and long-term outcome. However, the available therapeutic regimens of OA are merely symptom-relieving drugs unable to modify the progression of OA and to prevent long-term disability, and the symptom-structure discordance is well-recognized in clinical course of OA. Thus, the guidelines from the United States Food and Drug Administration (FDA) and the European Medicines Agency (EMA) point out that the effective disease-modifying osteoarthritis drugs (DMOADs) should be developed (Reginster et al., 2015; Oo et al., 2018). A DMOAD is expected a drug that modifies the underlying OA pathophysiology, thereby inhibiting structural damage to prevent or reduce long-term disability and offer potential symptomatic relief (Latourte et al., 2020). Currently, there are no US FDA- or EMA-approved DMOADs. But emerging treatments targeting inflammation, cartilage metabolism, and subchondral bone remodeling, which may retard the structural progression and induce disease remission, are promising to bring benefits to OA management in the future.

This narrative review will discuss recent developments of agents for the treatment of OA, including potential DMOADs and novel therapeutics for pain relief (Table 1). This review will focus more on drugs that have been in clinical trials, as well as attractive drugs with potential applications in preclinical research, to provide clinicians with recent advances in OA pharmacological therapies.

**TABLE 1:** Major emerging drugs to control structural damage and relieve pain in OA clinical trials.

Type of drug	Route of administration	Major findings	Stage of development	Clinical trials. gov identifier
Targeting inflammatory mechanisms				
IL-1 inhibitors				
Anakinra	Intra-articular	Anakinra did not significantly improve symptoms in patients with knee OA.	Phase II (knee OA)	NCT00110916
AMG 108	Subcutaneous/Intra-articular	AMG 108 showed statistically insignificant but numerically greater improvements in pain.	Phase II (knee OA)	NCT00110942
Canakinumab	Intra-articular	The clinical trial was completed, but the results have not been published.	Phase II (knee OA)	NCT01160822
Gevokizumab	Subcutaneous	The clinical trials were completed, but the results have not been published.	Phase II (erosive hand OA)	NCT01683396
			Phase II (erosive hand OA)	NCT01882491
Lutikizumab (ABT-981)	Subcutaneous	Lutikizumab was generally well tolerated in patients with knee OA and elicited an anti-inflammatory response.	Phase I (knee OA)	NCT01668511
		Lutikizumab did not improve pain or imaging outcomes in erosive hand OA compared with placebo.	Phase IIa (erosive hand OA)	NCT02384538
		Lutikizumab was not an effective analgesic/anti-inflammatory therapy in most patients with knee OA associated synovitis.	Phase IIa (knee OA)	NCT02087904 (ILL-USTRATE- K trail)
TNF-α inhibitors				
Etanercept	Subcutaneous	Subcutaneous injection of Etanercept for 24 weeks did not relieve pain effectively in patients with erosive hand OA compared with placebo.	—	NTR1192 (EHOA trail)
Infliximab	Intra-articular	Treatment with Infliximab can reduce the incidence of secondary OA in proximal interphalangeal joints in patients with active RA.	Exploratory observational longitudinal study	—
		Infliximab was safe, and significantly improved pain symptoms	Plot study (erosive hand OA)	—
Adalimumab	Subcutaneous	Adalimumab was not superior to placebo in relieving pain in patients with erosive hand OA.	Phase III (erosive hand OA)	NCT00597623
		Adalimumab did not affect synovitis or BMLs in patients with hand OA with MRI-detected synovitis.	—	ACTRN12612000791831 (HUMOR trial)
		Adalimumab significantly slowed the progression of joint aggressive lesions in a subpopulation with palpable tissue swelling of the interphalangeal joints.	—	EudraCT 2006–000925–71
DMARDs				
HCQ	Oral	HCQ did not relieve symptoms or delay structural damage.	—	ISRCTN91859104 (HERO trial)
MTX	Oral	MTX significantly reduced pain and improved synovitis in patients with symptomatic knee OA.	—	NCT01927484
		MTX added to usual care demonstrated significant reduction in knee OA pain at 6 months, and significant improvements in WOMAC stiffness and function. No effect on synovitis	Phase III (knee OA)	ISRCTN77854383 (PROMOTE trial)
		The clinical trial is ongoing	—	NCT03815448
Removing SnCs				
UBX0101	Intra-articular	The clinical trials were completed, but the results have not been published.	Phase I (knee OA)	NCT03513016
			Phase I (knee OA)	NCT04229225
			Phase II (knee OA)	NCT04129944
Curcuma longa extract	Oral	Curcuma longa extract was more effective than placebo for knee pain but did not affect knee effusion–synovitis or cartilage composition.	Phase II (knee OA)	ACTRN12618000080224
		The clinical trial is ongoing	Phase III (hip or knee pain)	NCT04500210
Targeting Cartilage Metabolism				
Wnt pathway inhibitors				
Lorecivivint (SM04690)	Intra-articular	Lorecivivint 0.07 mg was superior to the placebo in improving pain and function, and increased the JSW in patients with knee OA.	Phase I (knee OA)	NCT02095548
		Lorecivivint had no significant effects in knee OA patients, but significantly relieved pain, improved joint function, and increased JSW in a subgroup of patients (patients with unilateral symptomatic knee OA and unilateral symptomatic knee OA without extensive pain).	Phase IIa (knee OA)	NCT02536833
		The clinical trial is ongoing	Phase III (knee OA)	NCT03928184
Cathepsin-K inhibitors				
MIV-711	Oral	MIV-711 was not more effective than placebo for pain, but it significantly reduced bone and cartilage progression with a reassuring safety profile.	Phase Ⅱa (knee OA)	NCT02705625
MMP/ADAMTS inhibitors				
AGG-523	Oral	The clinical trials were completed, but the results have not been published	Phase I (knee OA)	NCT00454298
			Phase I (knee OA)	NCT00427687
M6495	Subcutaneous	The clinical trial was completed, but the results have not been published.	Phase Ib (knee OA)	NCT03583346
Growth factors				
Sprifermin (rhFGF18)	Intra-articular	Sprifermin appeared safe and well-tolerated, and it showed a statistically significant dose-dependent effect in reducing the loss of total and lateral femorotibial cartilage thickness and loss of lateral radiographic JSW.	Phase I (knee OA)	NCT01033994
		Sprifermin had a limited effect on pain improvement, but had a statistically significant effect in reducing the loss of total femorotibial cartilage thickness.	Phase II (knee OA)	NCT01919164 (FO-RWARD trial)
GEC-TGF-β1	Intra-articular	GEC-TGF-β1 significantly improved pain function and physical ability.	Phase II (knee OA)	NCT01221441
			Phase II (knee OA)	NCT01671072
		GEC-TGF-β1 had beneficial effects on pain and functional improvement in patients with OA, but had limited effects on structural improvement.	Phase III (knee OA)	NCT02072070
Activating AMPK pathway				
Metformin	Oral	Metformin may have a beneficial effect on long-term knee joint outcomes in those with knee OA and obesity.	Prospective cohort study (knee OA)	—
Targeting the Subchondral Bone				
Bisphosphonate				
Zoledronic Acid	Intra-articular	Zoledronic acid did not significantly reduce cartilage volume loss, relieve pain, or improve BMLs.	Phase Ⅲ (Knee OA)	ACTRN12613000039785
Calcitonin				
Salmon calcitonin	Oral	Salmon calcitonin did not improve pain symptoms and JSW in patients with symptomatic knee OA.	Phase Ⅲ (Knee OA)	NCT00486434
				NCT00704847
Strontium Ranelate	Oral	Strontium Ranelate significantly inhibited the narrowing of the medial femoral joint space, relieved pain, and improved physical function in patients with moderate to severe knee OA.	Phase Ⅲ (Knee OA)	ISRCTN41323372 (SEKOIA trial
Teriparatide	Subcutaneous	The clinical trial is ongoing.	Phase Ⅱ (knee OA)	NCT03072147
Vitamin D	Oral	Vitamin D supplementation, compared with placebo, did not result in significant differences in change in MRI-measured tibial cartilage volume or WOMAC knee pain score over 2 years, but might have beneficial effects on physical function, foot pain, depressive symptoms and effusion-synovitis.	Phase Ⅲ (Knee OA)	NCT01176344
Investigational Drugs to relieve pain				
NGF inhibitors				
Tanezumab	Subcutaneous	Tanezumab was significantly better than the placebo in improving pain and physical function, and PGA-OA.	Phase III (hip or knee OA)	NCT02697773
		Tanezumab statistically significantly improved pain, physical function and PGA-OA in patients with moderate to severe OA who had not responded to or could not tolerate standard-of-care analgesics	Phase III (hip or knee OA)	NCT02709486
Fasinumab	Subcutaneous	Fasinumab significantly improved pain and function in patients with OA, even in those who obtained little benefit from previous analgesics	Phase IIb/III (hip or knee OA)	NCT02447276
		The clinical trials are ongoing	Phase III (hip or knee OA)	NCT02683239
				NCT03285646
				NCT03161093
				NCT03304379
Triamcinolone acetonide sustained-release agent				
Zilretta (FX006)	Intra-articular	Zilretta significantly reduced ADP-intensity compared with saline-solution placebo. Zilretta significantly improved pain, stiffness, physical function, and the quality of life compared with both placebo and TAcs	Phase III (knee OA)	NCT02357459

OA: osteoarthritis; RA: rheumatoid arthritis; BMLs: bone marrow lesions; DMARDs: disease-modifying antirheumatic drugs; HCQ: hydroxychloroquine; MTX: methotrexate; WOMAC: Western Ontario and McMaster Universities Osteoarthritis Index; SnCs: senescent cells; JSW: joint space width; MMP: matrix metalloproteinase; ADAMTS: a disintegrin and metalloproteinase with thrombospondin motifs; rhFGF18: recombinant human fibroblast growth factor 18; NGF: nerve growth factor; PGA-OA: patient’s Global assessment of OA; ADP: average-daily-pain; TAcs: triamcinolone acetonide crystal suspensions.

## Investigational Drugs Targeting Inflammatory Mechanisms

The inflammatory mediators can be detected in both synovial fluid and serum in OA patients, indicating that inflammation does play a significant role in the pathogenesis of OA (LeGrand et al., 2001). OA is now seen as a low-grade inflammatory disease compared to rheumatoid arthritis (RA) (Scanzello and Loeser, 2015). Recently, studies have revealed that the low-grade, chronic, sterile inflammation associated with OA is closely related to dysregulation of the immune system as aging (Millerand et al., 2019). Anti-inflammatory therapeutics and treatment modalities targeting senescence processes may be promising approaches to attenuate disease progression of OA.

### Interleukin (IL)-1 Inhibitors

IL-1 has an increased expression in cartilage, synovium, and synovial fluid in OA patients (Sohn et al., 2012). It is an important proinflammatory cytokine and pain mediator resulting in pain sensitization, bone resorption, and cartilage destruction. Thus, IL-1 inhibitors may protect against structural changes in OA (Miller et al., 2014; Schett et al., 2016). Cytokines of the IL-1 family members include IL-1α, IL-1β, and endogenous IL-1 receptor antagonist (IL-1Ra). The ideal treatment is to effectively inhibit IL-1α and IL-1β without interfering with IL-1Ra.

1) Drugs targeting IL-1 receptor include human IL-1 receptor antagonist Anakinra, and human IL-1 receptor type 1 (IL-1R1) monoclonal antibody AMG 108 produced by genetic recombination technology. In two randomized, double-blind, placebo-controlled studies, it was found that subcutaneous (SC) or intravenous (IV) of AMG 108 and a single intra-articular (IA) injection of Anakinra were well tolerated (Chevalier et al., 2009; Cohen et al., 2011). Patients in the study received SC or IV injection of AMG 108 every 4 weeks for 12 weeks, and the results showed that patients who received AMG 108 showed statistically insignificant but numerically greater improvements in pain compared to placebo (Cohen et al., 2011). Similarly, IA injection of Anakinra did not significantly improve symptoms in patients with knee OA (Chevalier et al., 2009). Neither of these studies evaluated the effects on the joint structure.
2) Drugs targeting IL-1β include the humanized monoclonal antibody Canakinumab and the IL-1β allosteric modulating antibody Gevokizumab, which inhibit IL-1β receptor activation by tightly binding IL-1β. Canakinumab is considered as a disease-modifying antirheumatic drug (DMARD), has been shown to improve symptoms of juvenile idiopathic arthritis and RA, and decrease cartilage destruction (Sota et al., 2018). A recent preclinical study demonstrated that Canakinumab had protective effects on human OA chondrocytes *in vitro* (Cheleschi et al., 2015). In the Canakinumab Anti-inflammatory Thrombosis Outcome Study (CANTOS trial), it was observed that Canakinumab reduced not only cardiovascular events but also the incidence of total knee or hip replacement as a result of OA (Chevalier and Eymard, 2019). A phase II study (NCT01160822) on the safety, tolerability, pharmacokinetics, and pain effects of a single IA injection of Canakinumab in patients with knee OA was completed, but the results have not been published. Another phase II studies (NCT01683396; NCT01882491) to test the safety and biologic activity of Gevokizumab, and an open-label safety extension study of Gevokizumab (NCT02293564) in patients with hand OA were completed, but no published results are available.
3) Lutikizumab (formerly ABT-981) is a human dual variable domain immunoglobulin (DVD-Ig), simultaneously binding and inhibiting IL-1α and IL-1β (Lacy et al., 2015). In a randomized placebo-controlled phase I study, Lutikizumab was generally well tolerated in patients with mild to moderate knee OA, and significantly reduced serum concentrations of matrix metalloproteinase (MMP)-1 and high-sensitivity C-reactive protein (hsCRP) (Wang S. X. et al., 2017). However, the results from two recent phase II clinical studies to assess the efficacy of Lutikizumab in patients with hand OA and knee OA were unsatisfactory (Fleischmann et al., 2019; Kloppenburg et al., 2019). In erosive hand OA, Lutikizumab was administrated subcutaneously every 2 weeks for 26 weeks, but there were no significant differences in pain score, and in changes of X-ray or magnetic resonance imaging (MRI) scores between Lutikizumab and placebo (Kloppenburg et al., 2019). In knee OA with evidence of synovitis (ILLUSTRATE-K trial), Lutikizumab was administrated subcutaneously with three different doses (25, 100, and 200 mg) every 2 weeks for 50 weeks, the results showed that only lutikizumab 100 mg was slightly superior to the placebo in pain improvement at week 16 (Fleischmann et al., 2019). Moreover, at weeks 26 and 52, there were no significant differences between the lutikizumab and placebo groups in MRI-detected synovitis, radiographic medial and lateral joint space narrowing (JSN), and cartilage thickness (Fleischmann et al., 2019). These results suggest that IL-1 inhibition is not effective in most patients with OA. Whether subgroups of OA patients might have symptomatic or disease-modifying benefits from IL-1 inhibition remains an open question.

### Tumor Necrosis Factor-Alpha Inhibitors

TNF-α, a proinflammatory cytokine produced by synoviocytes and chondrocytes in OA, plays a central role in the induction of structural damage and pain modulation in OA. Besides, TNF-α enhances the production of a series of other proinflammatory cytokines (such as IL-6 and IL-8), stimulates the synthesis of MMP and cyclooxygenase (COX), and increases NO production (Orita et al., 2011). Preclinical studies suggested that anti-TNF-α therapy might exert a protective effect on articular cartilage by improving the structure of the subchondral bone and reducing cartilage matrix degradation (Ma et al., 2015). Thus, inhibitors of TNF-α might be considered as potential candidates for disease-modifying therapy in OA.

(1) Etanercept is a recombinant human tumor necrosis factor receptor type II antibody fusion protein. A study investigated the effect of IA injection of Etanercept for pain in moderate and severe knee OA. The results showed that compared with the hyaluronic acid group, direct injection of Etanercept into OA knee joints could effectively relieve the pain symptoms in OA patients (Ohtori et al., 2015). However, A recent randomized, double-blind, placebo-controlled trial (EHOA trial) found that the SC injection of Etanercept for 24 weeks did not relieve pain effectively in patients with erosive hand OA compared with placebo (Kloppenburg et al., 2018). In subgroup analysis, joints treated with Etanercept for 52 weeks showed more radiographic remodeling and less MRI bone marrow lesions (BMLs), which was more pronounced in actively inflamed joints at the baseline (Kloppenburg et al., 2018). In this study, Etanercept was observed to reduce serum levels of MMP-3, an important mediator of joint destruction (Kroon et al., 2020). Overall, this study did not provide evidence for the use of Etanercept to treat hand OA, but from a therapeutic strategy targeting inflammation, the authors believed that short-term treatment with TNF-α inhibitors during disease flares could be considered.
(2) Infliximab is a human/mouse chimeric monoclonal antibody of immunoglobulin G (IgG) 1/k subtype (composed of human IgG1 constant region and murine variable region). An exploratory observational longitudinal study found that treatment with Infliximab can reduce the incidence of secondary OA in proximal interphalangeal joints in patients with active RA (Guler-Yuksel et al., 2010). A pilot study investigated the efficacy and tolerability of IA injection of Infliximab in erosive hand OA (Fioravanti et al., 2009). The results showed that IA injection of Infliximab was safe, and significantly improved pain symptoms. Infliximab tended to reduce radiological scores of anatomical lesions in the hand, but the difference did not reach statistical significance. The study suggested a possible symptom- and disease-alleviating effect of Infliximab, but clinical trials are still needed to elucidate the true effect of Infliximab in OA.
(3) Adalimumab is the first bioengineered fully human monoclonal antibody that binds specifically to TNF and neutralizes the biological function of TNF by blocking its interaction with both Types 1 and 2 TNF receptors (TNF-R1 and -R2). A 12-month, double-blind, randomized controlled trial evaluated the efficacy and safety of Adalimumab in controlling structural damage in patients with erosive hand OA (Verbruggen et al., 2012). The tolerability and safety of Adalimumab in patients with erosive hand OA were similar to those in patients with other systemic rheumatic diseases. Compared with placebo, Adalimumab did not halt the progression of joint damage in overall patients, but it significantly slowed the progression of joint aggressive lesions in a subpopulation with palpable tissue swelling of the interphalangeal joints. However, in two randomized double-blind placebo-controlled trials, Adalimumab was not superior to placebo in relieving pain in patients with erosive hand OA (Chevalier et al., 2015; Aitken et al., 2018), and one study (HUMOR trial) also indicated that Adalimumab did not affect synovitis or BML in patients with hand OA with MRI-detected synovitis (Aitken et al., 2018).

### DMARDs

With the increasing acceptance of the inflammatory phenotype of OA, traditional DMARDs may have the potential to reduce pain and slow structural degeneration in OA. Hydroxychloroquine (HCQ) has been successfully used in the treatment of mild RA and other autoimmune diseases for many years (Ghouri and Conaghan, 2019). A randomized trial during 24 weeks showed that compared with placebo, HCQ was not effective in reducing the symptoms of hand OA (Lee et al., 2018). Recently, a randomized double-blind placebo-controlled trial (HERO trial) with 12-month follow-up evaluated the efficacy of HCQ in hand OA patients with moderate to severe pain, and the results showed that HCQ did not relieve symptoms or delay structural damage (Kingsbury et al., 2018).

Methotrexate (MTX) is a traditional DMARD for the treatment of some autoimmune diseases such as RA. The study (NCT01927484) reported that oral MTX significantly relieved pain and reversed features of synovitis in patients with symptomatic knee OA, which indicated MTX as an option for the treatment of knee OA (Abou-Raya et al., 2018). A pragmatic phase III RCT was completed (PROMOTE trial) to determine whether oral MTX reduced pain and synovitis associated with knee OA in 2019 (Kingsbury et al., 2015). The results presented at Osteoarthritis Research Society International (OARSI) Annual Congress showed that MTX significantly reduced knee OA pain, and significantly improved Western Ontario and McMaster Universities Osteoarthritis Index (WOMAC) scores for stiffness and function. However, MTX did not change the synovial volume assessed by MRI in this study. Meanwhile, a multicenter RCT study to investigate the effect of oral MTX on pain and synovitis in patients with mid-to late-stage knee OA (NCT03815448) is ongoing (Zhu et al., 2020), and further data are expected to come soon. Overall, more evidence is needed to clearly define the role of MTX in OA treatment.

### Targeting Senescent Cells

The innate immune activation caused by the dysregulation of the immune system with aging is considered to play a crucial role in the chronic inflammation of OA (Jeon et al., 2018). Age-related mitochondrial dysfunction and associated oxidative stress might induce senescence in joint tissue cells (Coryell et al., 2020). The accumulation of SnCs in joints causes the secretion of pro-inflammatory and pro-catabolic factors (cytokines, chemokines, MMPs), which is called a “senescence-associated secretory phenotype” (SASP) (Childs et al., 2017; Millerand et al., 2019). Direct targeting the SnCs provides a potential opportunity to eliminate the source of OA disease (Childs et al., 2017; Jeon et al., 2017). UBX0101 is a small molecule lysosomal agent that can reduce the expression of SASP factors and improve overall joint function (Jeon et al., 2017). Currently, several randomized, placebo-controlled clinical trials of UBX0101 are all completed in 2020 to evaluate the efficacy, safety, and tolerability of IA injection of UBX0101 in knee OA patients (NCT03513016, NCT04229225, and NCT04129944), and the results will be released soon.

### Curcuma Longa Extract

Curcuminoids, are the principal extracted from the CL root (Family Zingiberaceae), which comprise curcumin, demethoxycurcumin (DMC) and bisdemethoxycurcumin (BDMC) (Cao et al., 2014). The curcumin is the main active and effective ingredient. Curcumin is known to suppress oxidative stress and inflammation by scavenging active oxygen and inhibiting nuclear factor-kappa β (NF-κβ) pathway (Shen and Ji, 2012; Wang J. et al., 2017). A systematic review and meta-analysis of RCT enrolled 797 patients with primarily knee OA demonstrated that Curcuminoids had some beneficial effects on knee pain and quality of life in patients with knee OA (Onakpoya et al., 2017). Recently, a single-center, randomized, placebo-controlled trial with 12-week follow-up evaluated the efficacy of CL in patients with symptomatic knee OA and effusion-synovitis, and the results showed that CL was superior to placebo in relieving knee pain but did not affect the effusion-synovitis volume or cartilage composition as assessed by MRI (Wang et al., 2020). However, the follow-up time was relatively short so that it might be insufficient to detect a change in the cartilage- and synovium-specific outcomes in this study. Another double-blind, randomized, parallel-group, phase III comparative study (NCT04500210) of CL and placebo to patients with mild to moderate OA of the knee and or hip is still recruiting. Further researches with larger sample sizes are needed to assess the clinical significance of CL in OA treatment.

## Investigational Drugs Targeting Cartilage Metabolism

The characteristic sign of OA is cartilage destruction, so emerging drugs targeting the molecular mechanism of articular cartilage should be an attractive therapeutic strategy for OA. The research direction is mainly to delay cartilage destruction by anti-catabolic agents and stimulate cartilage development and repair by anabolic agents.

### Wnt Signaling Pathway Inhibitors

The balance of Wnt pathway activity is integral for regulating the differentiation of progenitor cells in the joint and maintaining cartilage homeostasis (Lories et al., 2013; Thysen et al., 2015). In OA, aberrant Wnt pathway activity leads to the differentiation of progenitor cells into osteoblasts while chondrocyte development is blocked, as well as the increased secretion of catabolic enzymes and inflammation.

Preclinical studies demonstrated that Wnt pathway inhibitors could delay the development of OA in animal models; however, excessive inhibition, in turn, caused cartilage and bone destruction. Thus, targeting the Wnt pathway and controlling it within an optimal range is a potential therapeutic avenue (Usami et al., 2016; Deshmukh et al., 2018).

Lorecivivint (formerly SM04690) is a small-molecule Wnt pathway inhibitor and modulates the Wnt pathway by inhibiting two intranuclear targets, intranuclear kinases CDC-like kinase 2 (CLK2) and dual-specificity tyrosine phosphorylation-regulated kinase 1 A (DYRK1A) (Deshmukh et al., 2019). In a 24-week, randomized, placebo-controlled phase I study, a single IA injection of Lorecivivint (0.03, 0.07, or 0.23 mg) appeared safe and well-tolerated (Yazici et al., 2017). Lorecivivint 0.07 mg was superior to the placebo in improving WOMAC pain scores and function scores in patients with moderate to severe knee OA, while the 0.07 mg dose group also showed an increase from baseline in radiographic joint space width (JSW) (Yazici et al., 2017).

Recently, the results of a 52-week multicenter, randomized, double-blind, placebo-controlled phase IIa study announced that Lorecivivint treatment was not superior to placebo for improving pain, joint function, and radiographic JSW in patients with moderate to severe knee OA (Deshmukh et al., 2019), but in subgroup patients with unilateral symptomatic knee OA or unilateral symptomatic knee OA without extensive pain, Lorecivivint 0.07 mg significantly relieved pain, improved joint function, and increased JSW compared with placebo (Deshmukh et al., 2019). The study suggested that Lorecivivint might be effective in OA patients with a certain phenotype.

Besides, a phase III clinical study (NCT03928184) has been initiated in 2019 to assess the long-term efficacy and safety of Lorecivivint in the treatment of knee OA, and Lorecivivint has the potential to be an effective treatment for OA.

### Cathepsin-K Inhibitors

Cathepsin-K is the predominant cysteine cathepsin in the skeleton and it plays an important role in the resorption of cartilage and bone (Dejica et al., 2008). Several observations have demonstrated up-regulation of cathepsin K in OA cartilage and inflamed synovial tissue (Salminen-Mankonen et al., 2007). Cathepsin-K may be an attractive therapeutic target for diseases with excessive bone resorption such as osteoporosis and OA. Cathepsin K inhibitors have shown structural protection and analgesic effects in animal models of joint degeneration (Lindström et al., 2018a; Nwosu et al., 2018).

The results of phase II clinical study evaluating the efficacy and safety of the Cathepsin-K inhibitor Balicatib in OP and OA patients showed that it could improve bone mineral density in OP patients, but it failed to decrease cartilage volume loss (CVL) in patients with knee OA (Duong et al., 2016). Also, Balicatib could lead to dose-related adverse effects-Morphea-like skin reactions (Runger et al., 2012).

MIV-711 is a highly selective cathepsin K inhibitor that has been shown in preclinical animal models of OA to reduce cartilage lesions, reduce levels of biomarkers reflecting the degradation of bone and cartilage [carboxy-terminal collagen cross links (CTX)-I and CTX-II] and prevent subchondral bone loss (Lindström et al., 2018a; Lindström et al., 2018b). A recent randomized, double-blind, placebo-controlled phase IIa study to assess the efficacy and safety of MIV-711 in symptomatic patients with Kellgren-Lawrence (KL) grade 2 and 3 knee OA (Conaghan et al., 2020). The results showed that oral administration of MIV-711 (100 mg/d or 200 mg/d) for 26 weeks had a significant protective effect on both bone and cartilage structures, and significantly reduced the levels of CTX-I and CTX-II, but failed to meet the primary study endpoint of alleviating knee joint pain (Conaghan et al., 2020). MIV-711 has a good safety profile, but its clinical efficacy remains to be validated in longer-term and larger-scale clinical studies.

### MMP/ADAMTS Inhibitors

Aggrecan and type II collagen are two main components of articular cartilage, which are essential for maintaining the function and integrity of cartilage (Malfait and Tortorella, 2019). Aggrecan provides the compressibility of cartilage, while collagen provides its elasticity. These macromolecules are decomposed by proteolysis. MMPs and aggrecanase (a disintegrin and metalloproteinase with thrombospondin motifs (ADAMTS), mainly ADAMTS-4 and ADAMTS-5) are demonstrated to have critical roles in the degradation of type II collagen and aggrecan, respectively, and are considered as potential targets for OA treatment.

(1) In preclinical trials, highly selective MMP-13 inhibitors (such as ALS1-0635 and PF152) have shown advantages in slowing the progression of OA (Piecha et al., 2010; Schnute et al., 2010). However, the available data on the role of MMP-13 inhibitors in OA treatment is limited, and human clinical trials are still needed to observe the efficacy of MMP-13 inhibitors as DMOADs.
(2) At present, the investigational drugs targeting ADAMTS-5/ADAMTS-4 include a chimeric murine/human ADAMTS-5 monoclonal antibody-CRB0017, which was reported to slow OA disease progression after IA administration in animal models of OA (Chiusaroli et al., 2013), and a humanized ADAMTS-5-selective monoclonal antibody, GSK2394002, which was reported to have structural modification and analgesic effects in animal models of OA (Larkin et al., 2015; Miller et al., 2016). AGG-523, an orally small molecule inhibitor of ADAMTS-4 and ADAMTS-5, was the first to enter the human phase I study (NCT00454298 and NCT00427687), but these trials were discontinued for unknown reasons. M6495, a novel anti-ADAMTS-5 inhibiting Nanobody, showed dose-dependent protection against cartilage deterioration in *ex vivo* cartilage cultures (Siebuhr et al., 2020). A phase Ib (NCT03583346) clinical trial to assess safety, tolerability, immunogenicity, pharmacokinetics, and pharmacodynamics of SC injections of M6495 in knee OA patients was completed in 2019, but the results have not yet been published.

### Growth Factors

Different from using anti-catabolic agents to delay the progression of cartilage destruction, an alternative approach is to stimulate the growth and repair of cartilage for the treatment of OA. Several growth factors have been shown to stimulate cartilage anabolism and promote cartilage repair *in vitro* and animal models of OA. Growth factors may have potential therapeutic effects on OA.

(1) Sprifermin is a recombinant human fibroblast growth factor 18 (rhFGF18) (Onuora, 2014), and preclinical data had shown that Sprifermin bound to and activated fibroblast growth factor receptor 3 (FGFR3) in cartilage to promote chondrogenesis, cartilage matrix formation, and cartilage repair *in vivo* and *in vitro* (Moore et al., 2005; Gigout et al., 2017; Reker et al., 2017; Sennett et al., 2018). A randomized, double-blind, placebo-controlled phase I b proof-of-concept trial evaluated the efficacy and safety of IA injection of Sprifermin (10, 30, and 100 μg) in patients with symptomatic knee OA (Lohmander et al., 2014). The results showed that Sprifermin appeared safe and well-tolerated. Although Sprifermin was not superior to placebo in reducing the loss of central medial femorotibial compartment (cMFTC) cartilage thickness and improving pain, it showed a statistically significant dose-dependent effect in reducing the loss of total and lateral femorotibial cartilage thickness and loss of lateral radiographic JSW (Lohmander et al., 2014). Two post-hoc analyses of this study demonstrated that Sprifermin (100 µg) reduced cartilage loss, increased cartilage thickness, and improved BMLs (Eckstein et al., 2015; Roemer et al., 2016).

A 5-years, dose-finding, multicenter phase II clinical trial (FORWARD trial), published in 2019, showed that IA injection of 100 μg Sprifermin every 6 or 12 months significantly increased the total femorotibial joint cartilage thickness in patients with symptomatic knee OA after 2 years, and this effect was dose-dependent. Sprifermin had a limited effect on pain improvement in this study (Hochberg et al., 2019). Recently, two post-hoc exploratory analyses were carried out on this study, and the results showed that sprifermin treatment could significantly increase cartilage thickness and reduce cartilage loss, making cartilage loss in patients with knee OA similar to that of healthy subjects (Brett et al., 2020; Eckstein et al., 2020). The above studies supported the conclusions that sprifermin modified structural progression and could be a potential DMOAD.

(2) Transforming growth factor-β1 (TGF-β1) plays an important role in the development and maturation of articular cartilage and the phenotypic maintenance of chondrocytes (Yang et al., 2001; Crane et al., 2016). The expression of TGF-β1 in healthy cartilage is significantly higher than that in OA cartilage; however, it has been found that overexpression of TGF-β1 leads to OA-like changes in the knee joint of C57Bl/6 mice, including hyperplasia of the synovium and osteophyte formation (Bakker et al., 2001). Recently, Liu et al. demonstrated that TGF-β had different effects on human OA mesenchymal stromal cells (OA-MSC) and chondrocytes (OAC). While TGF-β stimulated chondrogenesis in OAC, it induced hypertrophy, mineralization, and MMP-13 in OA-MSC (Liu et al., 2020).

SB-505124 is a TGF-β type I receptor inhibitor, and it was found *in vitro* and in animal models of OA that TGF-β1 overexpression in osteoclasts was responsible for chondrocyte apoptosis and cartilage degeneration in OA, and SB-505124 could inhibit the degradation of articular cartilage (Zhang et al., 2018).

Tissue Gene-c (TG-C) is a cell-mediated gene therapy that delivers allogeneic chondrocytes expressing TGF-β1 directly to the damaged knee joint, consisting of irradiated allogeneic human

chondrocytes that express TGF-β1 and normal allogeneic human chondrocytes in a 1:3 ratio (GEC-TGF-β1) (Ha et al., 2012). Two randomized, double-blind, placebo-controlled phase II studies to evaluate the safety and efficacy of IA injection of GEC-TGF-β1 in patients with knee OA (Cherian et al., 2015; Ha et al., 2015). The results showed that most of the adverse events were local reactions and did not require further treatment, and only a small number of patients had allergic reactions but recovered within 24 h. Moreover, compared with the placebo, GEC-TGF-β1 could significantly improve pain and physical function. However, neither of these studies evaluated the effect of GEC-TGF-β1 on cartilage regeneration and OA imaging changes. The results of a phase III trial (NCT02072070) suggested that GEC-TGF-β1 had beneficial effects on pain and functional improvement in patients with OA, but had limited effects on structural improvement (Kim et al., 2018).

### Metformin

Metformin is a safe and well-tolerated oral biguanide that has been used as the first-line therapy for type 2 diabetes for more than 50 years. Preclinical studies had shown that Metformin could significantly attenuate articular cartilage degeneration and relieve pain in the OA mouse model (Li H. et al., 2020). Besides, it was found that the chondroprotective effect of metformin was mediated by activation of adenosine monophosphate-activated protein kinase (AMPK) signaling. Metformin could enhance AMPK expression and phosphorylation in chondrocytes, and increase the production of type II collagen and reduce the level of MMP-13 by activating AMPK pathway (Li J. et al., 2020). A nationwide, retrospective, matched-cohort study evaluated 968 patients with OA and type 2 diabetes mellitus (T2DM) during 10 years of follow-up and the results showed that OA patients with T2DM under combination COX-2 inhibitors and Metformin therapy were associated with lower joint replacement surgery rates than COX-2 inhibitors only (Lu et al., 2018). Recently, a prospective cohort study reported that metformin had a beneficial effect on long-term knee outcomes in obese knee OA patients, and metformin significantly reduced the loss of medial knee cartilage volume (Wang et al., 2019). Currently, randomized controlled trials are still needed to confirm these findings and to determine whether metformin can be considered as a potential disease-modifying drug for knee OA with or without obese phenotype.

## Investigational Drugs Targeting the Subchondral Bone

Increased subchondral bone resorption and bone turnover contribute to the pathogenesis of OA (Karsdal et al., 2014). Thus, the subchondral bone may be a potential target for OA therapy. However, currently available agents targeting the subchondral bone haven't been approved for the treatment of OA due to the inconsistent efficacy or safety considerations, including Zoledronic Acid, Calcitonin, and Strontium ranelate.

### Bisphosphonate

One small randomized clinical trial stated that intravenous Zoledronic Acid was beneficial in improving pain and BMLs in knee OA patients at 6 months (Vaysbrot et al., 2018). BMLs detected by MRI represented areas of high bone turnover and active bone remodeling, and bisphosphonates might be beneficial for patients with high metabolic activity (Kuttapitiya et al., 2017). However, recently, a 24-month multicenter, double-blind placebo-controlled randomized clinical trial assessed the effects of twice-yearly intravenous Zoledronic Acid for 24 months on CVL in patients with symptomatic knee OA and BMLs (Cai et al., 2020). The results showed that Zoledronic Acid did not significantly reduce cartilage volume loss, relieve pain, or improve BMLs. These findings did not support intravenous Zoledronic Acid to treat knee OA. A randomized, double-blind, parallel-group, multicenter, placebo-controlled, dose-ranging study (EudraCT2018-002081-39) to assess the efficacy and safety of IA injection of clodronate for knee OA is currently ongoing, and no results are available.

### Calcitonin

A combined reporting of two randomized, double-blind, multi-center, placebo-controlled trials (NCT00486434 and NCT00704847) that included 1176 and 1030 patients, respectively, showed that oral salmon calcitonin (sCT) for 24 months did not improve pain symptoms and joint space width (JSW) measured by X-ray in patients with symptomatic knee OA (Karsdal et al., 2015).

### Strontium Ranelate

Strontium Ranelate is indicated for the treatment of postmenopausal osteoporosis (Han et al., 2017). Preclinical studies indicated that it reduced subchondral bone resorption and stimulated cartilage matrix formation *in vitro* and in rat OA model (Coulombe et al., 2004; Tat et al., 2011; Yu et al., 2013). A 3-year multicenter, randomized, double-blind, placebo-controlled Phase III clinical trial (SEKOIA trial) showed that Strontium Ranelate significantly inhibited the narrowing of the medial femoral joint space, relieved pain, and improved physical function in patients with moderate to severe knee OA compared with placebo (Reginster et al., 2013). A post hoc analysis of the SEKOIA trial found that Strontium Ranelate was also significantly associated with decreased MRI-assessed CVL and BMLs (Pelletier et al., 2015). However, although Strontium Ranelate has a significant protective effect on the joint structure and clinically relevant improvement of symptoms of knee OA, the use of Strontium Ranelate in OA is limited by its cardiovascular risk, particularly the side effects of thromboembolism.

### Teriparatide

Teriparatide is a recombinant human parathyroid hormone (PTH), derived from the 1–34 amino acid fragment of human PTH (Oo and Hunter, 2019). It promotes the proliferation and survival of osteoblasts, which is a bone anabolic therapy for osteoporosis (Sampson et al., 2011). A preclinical study showed that Teriparatide could decelerate cartilage degeneration and induced cartilage matrix regeneration in mice administered a meniscal/ligamentous knee injury (Macica et al., 2011). Teriparatide may become a novel candidate therapy for injury-induced OA. A phase II study (NCT03072147) to assess the chondroregenerative efficacy and safety of Teriparatide for knee OA is still ongoing, and the estimated study completion date is in 2022.

### Vitamin D

A prospective study determined that sunlight exposure and serum 25(OH)D levels were both positively associated with knee cartilage volume in older people, suggesting that vitamin D is an important hormonal contributor to cartilage homeostasis (Ding et al., 2009). Thus, Vitamin D supplementation potentially prevented the progression of OA. However, A 2-year RCT showed that Vitamin D supplementation at a dose sufficient to elevate serum levels of 25-hydroxyvitamin D to >36 ng/ml did not reduce knee pain or CVL in patients with symptomatic knee OA (McAlindon et al., 2013). A multicenter randomized, double-blind, placebo-controlled clinical trial (VIDEO trial) evaluated the effects of vitamin D supplementation in patients with symptomatic knee OA and low serum 25-hydroxyvitamin D levels (Jin et al., 2016). The results showed that Vitamin D supplementation did not prevent tibial cartilage loss or relieve knee pain over 2 years, but improved physical function (Jin et al., 2016) and reduced joint effusion synovitis (Wang X. et al., 2017). Three post-hoc exploratory analysis were carried out on the VIDEO trial. Vitamin D supplementation and maintaining vitamin D sufficiency (25-hydroxyvitamin D > 50 nmol/L at month 3 and 24) over 24 months might be benefificial for depressive symptoms (Zheng et al., 2019) and foot pain (assessed by manchester foot pain and disability index) (Tu et al., 2020) in patients with knee OA. Maintaining vitamin D sufficiency significantly reduced tibial cartilage volume loss and effusion-synovitis volume, and improved physical function compared with those who did not (Zheng et al., 2017).

## Investigational Drugs to Relieve Pain

NSAIDs and opioid drugs are primary pharmacological treatments for pain palliation in OA. But these medications are unsuitable for long-term use because of side effects, and their roles in pain control are limited (McAlindon and Bannuru, 2010; Zhang et al., 2010). Patients with OA continue to suffer from inadequate pain relief. Thus, although the development of drugs that can reverse the structural progression of joint damage in OA is important, it is still necessary to consider the effect of drugs against pain (Karsdal et al., 2016; Miller et al., 2018). Besides, there is also an urgent need to develop new ideal therapies, which are safe, simple, long-acting, and convenient to treat the chronic pain associated with OA.

### Monoclonal Antibodies Neutralizing Nerve Growth Factor

NGF is a neurotrophin that stimulates the growth of nociceptive nerve fibers and the expression of nociceptive cell surface receptors (Denk et al., 2017; Vincent, 2020). Almost all structures in the joint are innervated with nociceptive nerve fibers, and elevated NGF levels may be sources of refractory knee pain in OA (Malfait and Schnitzer, 2013; Denk et al., 2017). NGF is therefore an attractive target for novel analgesic agents. Tanezumab, Fulranumab, and Fasinumab are monoclonal antibodies that specifically target NGF and inhibit binding to its receptors (Ghouri and Conaghan, 2019). Tanezumab is the most widely studied and has completed pivotal phase III clinical trials, and Fasinumab is in the midst of phase III clinical trials (NCT02683239, NCT03285646, NCT03161093, and NCT03304379), while Janssen has discontinued the clinical development of Fulranumab, with no active trials being underway (Cao et al., 2020). The US FDA recently has granted fast-track certification (a process designed to facilitate the development and expedite the review of new therapies to treat serious conditions and fill unmet medical needs) for Tanezumab for the treatment of chronic pain in patients with OA or chronic low back pain, and Tanezumab is expected to be approved for clinical use soon.

A meta-analysis of 10 randomized controlled trials enrolled 7,665 patients demonstrated that Tanezumab was superior to placebo in pain relief and improvement in physical function and patient’s global assessment (PGA) in knee and hip OA patients (Chen J. et al., 2017). A phase IIb/III clinical trial assessed the efficacy, tolerability, and joint safety of Fasinumab in patients with hip and/or knee OA (Dakin et al., 2019). The results showed that Fasinumab significantly improved pain and function in patients with OA, even in those who obtained little benefit from previous analgesics (Dakin et al., 2019). A phase III clinical trial evaluated 696 patients with hip and/or knee OA who had not responded to or were unable to receive standard analgesics (Schnitzer et al., 2019). Patients received by 2 SC injections of Tanezumab (2.5 mg administered at baseline and week 8 or 2.5 mg administered at baseline and 5 mg at week 8) or placebo at day 1 and week 8. The results showed that Tanezumab was significantly better than the placebo in improving scores assessing pain and physical function, and PGA-OA (Schnitzer et al., 2019). Recently, another phase III clinical trial evaluated 849 patients with hip and/or knee OA who had not responded to or could not tolerate standard-of-care analgesics. Patients received SC Tanezumab 2.5 mg or 5 mg or placebo every 8 weeks (Berenbaum et al., 2020). The results showed that Tanezumab 5 mg statistically significantly improved pain, physical function and PGA, and Tanezumab 2.5 mg significantly improved pain and physical function, but did not improve PGA (Berenbaum et al., 2020).

It should be noted that anti-NGF treatment may lead to treatment-related rapidly progressive OA (PROA) and osteonecrosis (Hochberg, 2015). These serious joint-related adverse events drove the FDA to place a partial clinical hold on NGF antibodies. By reviewing the adverse events reported in clinical trials, it was found a dose-response relationship between osteonecrosis and NGF antibodies, with the dose of Tanezumab ranging from 2.5 to 10 mg and the dose of Fasinumab ranging from 3 to 9 mg (Hochberg, 2015; Lane and Corr, 2017; Dakin et al., 2019). Therefore, the maximum dose of Tanezumab was reduced to 5 mg after resuming the clinical trials in 2015. Importantly, compared with Tanezumab monotherapy, Tanezumab combined with NSAIDs treatment appeared to increase the risk of RPOA (Hochberg et al., 2016). It seemed that more joint replacements had been observed in patients treated with Tanezumab, but most were personal choices and not associated with adverse events (Schnitzer et al., 2019).

The anti-NGF treatment undoubtedly provides great potential for improving the pain and function of patients with severely symptomatic OA, but it carries the risk of aggravating the structural progression of OA (Miller et al., 2017). Therefore, in addition to using the lowest effective dose to mitigate the risk, it is essential to identify the patient population most suitable for this therapeutic approach. Jayabalan and Schnitzer believed that individuals with preexisting joint abnormalities, such as subchondral insufficiency fractures, who were at increased risk for PROA when treated with anti-NGF, should not be considered for the anti-NGF treatment. On the other hand, anti-NGF may be a particularly useful drug for specific populations for whom NSAIDs are contraindicated and/or not recommended (Jayabalan and Schnitzer, 2017).

### Intra-articular Corticosteroid

#### Triamcinolone Acetonide Sustained-Release Agent

Triamcinolone acetonide (TA) is an intra-articular corticosteroid to relieve pain, but its magnitude of benefit rapidly wanes post-injection for rapid systemic absorption (Kraus et al., 2018). Zilretta (formerly FX006) is a novel type of extended-release TA formulation in 75:25 poly microsphere, which is designed to prolong TA residence in the joint compared with standard TA crystal suspensions (TAcs) (Conaghan et al., 2018a). A phase III, multicenter, double-blind, randomized controlled trial compared FX006 (32 mg), TAcs (40 mg), and saline placebo in 484 patients with knee OA (Conaghan et al., 2018b). Although FX006 did not significantly reduce the average-daily-pain (ADP)-intensity of OA compared to TAcs at 12 weeks, it reached the primary endpoint of a significant improvement in ADP-intensity compared with placebo. In addition, FX006 significantly improved Western Ontario and McMaster Universities Osteoarthritis Index (WOMAC) scores for pain, stiffness, and physical function, and Knee Injury and Osteoarthritis Outcome Score Quality of Life (KOOS-QOL) scores for the quality of life at 12 weeks compared with both placebo and TAcs (Conaghan et al., 2018b). FX006 causes less blood glucose elevation compared to standard TAcs in type 2 diabetic patients. For this reason, FX006 has been licensed by the FDA in October 2017 for the treatment of OA-related knee pain.

#### IA Triamcinolone

A two-year, randomized, placebo-controlled, double-blind trial (NCT01230424) compared Triamcinolone (40 mg), and saline placebo in 140 patients with symptomatic knee OA. The results showed that IA Triamcinolone every 3 months for 2 years significantly increased CVL and did not improve knee pain (McAlindon et al., 2017). These findings do not support this long-term treatment for patients with symptomatic knee OA.

## Expert Opinion

OA is a chronic, painful and disabling arthritis with significant burden on the individual and society. With the population aging and obesity, the incidence of OA is increasing as a leading cause of disability worldwide (Peat and Thomas, 2020). To date, no effective drug is able to inhibit the structural damage or reduce long-term disability, or relieve pain with an acceptable benefit-to-risk profile in OA (Latourte et al., 2020). For these reasons, the OARSI led an effort to submit a White Paper to the FDA in support of the designation of OA as a serious disease in 2016. Actually, OA is a severe disease as RA for their similar disability rates, morbidity, costs, and increased mortality rates (Pincus et al., 2019). In the past few years, it has been realized that a complex interaction of multifactorial mechanisms is involved in the pathophysiology of OA. The heterogeneous condition of OA determines that there is no miracle therapeutic strategy fitting for all patients. Also, this heterogeneity may be the major cause for the failure of clinical trials testing therapeutics intended for structure modification or symptom relief in OA.

Various OA phenotypes and endotypes have been explored to overcome this barrier (Deveza et al., 2019), such as synovial inflammatory phenotype, osteoporotic phenotype, articular cartilage degradation phenotype, metabolic phenotype and so on. However, there are few clinical trials to stratify patients based on these phenotype-guided approaches yet. OA phenotyping would be helpful to therapy selection and expedite the development of investigational tailored drugs directly toward variable courses of OA. Metabolomic studies and innovative machine learning approaches may greatly help to determine the key variables to differentiate specific OA subgroups and progression phenotypes (Carlson et al., 2019; Nelson et al., 2019). Nelson et al. observed that baseline variables as BMLs, osteophytes, medial meniscal extrusion, and urine CTX-II were useful to identify progression OA phenotypes at 48 months, while WOMAC pain, lateral meniscal extrusion, and serum N-terminal pro-peptide of collagen IIA (PIIANP) were associated with non-progression phenotypes (Nelson et al., 2019). Establishing OA phenotypes and then setting up distinctive outcome measures for each phenotype is a way to organize more effective and stratified clinical trials in OA in future (Roman-Blas et al., 2020). For example, the synovitis features detected by MRI or ultrasound (US) have the potential to become the useful outcome measures and could be used in clinical trials of new drugs that target synovitis in OA patients with inflammatory phenotype.

To identify the patient population with disease progression is vital to appropriately power clinical trials. The OA patients in the progressed periods are potentially more responsive to interventions, and these patients might be recruited in DMOAD trials to assess the efficacy of a new drug in the future. Sensitive and valid biomarkers are expected to become useful tools to predict OA progression and understand mechanisms of progression (Roman-Blas et al., 2020). On the other hand, OA may only be retarded at early to mid-stages instead of established or advanced OA. To identify the patient population in the early to mid-stages of the disease is also important. Some studies have proposed using MRI or US for the test of disease-modifying approaches and recruiting patients with early diseases as defined on MRI or US in clinical trials (Eckstein and Le Graverand, 2015; Wang et al., 2021).

There is an unmet need for DMOADs. One approach to develop such drugs is to use imaging-assessed joint structural changes such as loss of cartilage volume/thickness, BMLs and synovitis as primary endpoints. However, these endpoints have not been formally accepted by drug administrations. Recently, several authors from The United States Food and Drug Administration proposed a composite endpoint such as “time to total knee replacement (TKR) or severe pain or severely impaired functioning” which can substantially reduce sample size compared to the use of TKR alone (Kim et al., 2020). The endpoints such as this based on direct measures of patients’ functions, feels or survive would be more clinically relevant for development of OA drugs.

**FIGURE 1:**
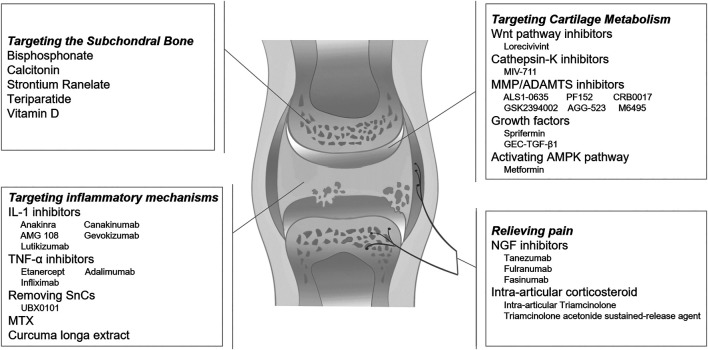
Potential pharmacological therapies for osteoarthritis.

A variety of potential therapeutics targeting on inflammation, cellular senescence, cartilage metabolism, subchondral bone remodeling, and peripheral nociceptive pathway are expected to reshape the landscape of OA treatment over the next few years (Figure 1). The cartilage destruction is the main characteristic sign of OA. Novel agents targeting articular cartilage molecular mechanisms seem to be most promising. Lorecivivint, MIV-711 and Sprifermin are promising agents as DMOADs to slow disease progression. Long-term RCTs are still needed to confirm the safety and efficacy of these novel OA pharmacotherapy medicines.

## References

[B1] Abou-Raya A. Abou-Raya S. Khadrawe T. (2018). Retracted: methotrexate in the treatment of symptomatic knee osteoarthritis: randomised placebo-controlled trial. Ann. Rheum. Dis. 77 (7), e46. 10.1136/annrheumdis-2013-204856 24675098

[B2] Aitken D. Laslett L. L. Pan F. Haugen I. K. Otahal P. Bellamy N. (2018). A randomised double-blind placebo-controlled crossover trial of HUMira (adalimumab) for erosive hand OsteoaRthritis - the HUMOR trial. Osteoarthritis Cartilage 26 (7), 880–887. 10.1016/j.joca.2018.02.899 29499287

[B3] Arden N. K. Perry T. A. Bannuru R. R. Bruyère O. Cooper C. Haugen I. K. (2020). Non-surgical management of knee osteoarthritis: comparison of ESCEO and OARSI 2019 guidelines. Nat. Rev. Rheumatol. 17, 59. 10.1038/s41584-020-00523-9 33116279

[B4] Bakker A. C. van de Loo F. A. J. van Beuningen H. M. Sime P. van Lent P. L. E. M. van der Kraan P. M. (2001). Overexpression of active TGF-beta-1 in the murine knee joint: evidence for synovial-layer-dependent chondro-osteophyte formation. Osteoarthritis Cartilage 9 (2), 128–136. 10.1053/joca.2000.0368 11237660

[B5] Bally M. Dendukuri N. Rich B. Nadeau L. Helin-Salmivaara A. Garbe E. (2017). Risk of acute myocardial infarction with NSAIDs in real world use: bayesian meta-analysis of individual patient data. BMJ 357, j1909. 10.1136/bmj.j1909 28487435 PMC5423546

[B6] Berenbaum F. Blanco F. J. Guermazi A. Miki K. Yamabe T. Viktrup L. (2020). Subcutaneous tanezumab for osteoarthritis of the hip or knee: efficacy and safety results from a 24-week randomised phase III study with a 24-week follow-up period. Ann. Rheum. Dis. 79 (6), 800–810. 10.1136/annrheumdis-2019-216296 32234715 PMC7286052

[B7] Brett A. Bowes M. A. Conaghan P. G. Ladel C. Guehring H. Moreau F. (2020). Automated MRI assessment confirms cartilage thickness modification in patients with knee osteoarthritis: post-hoc analysis from a phase II sprifermin study. Osteoarthritis Cartilage 28 (11), 1432–1436. 10.1016/j.joca.2020.08.005 32860991

[B8] Cai G. Aitken D. Laslett L. L. Pelletier J.-P. Martel-Pelletier J. Hill C. (2020). Effect of intravenous zoledronic acid on tibiofemoral cartilage volume Among patients with knee osteoarthritis with bone marrow lesions. JAMA 323 (15), 1456–1466. 10.1001/jama.2020.2938 32315057 PMC7175085

[B9] Cao Y. Xu R. X. Liu Z. (2014). A high-throughput quantification method of curcuminoids and curcumin metabolites in human plasma via high-performance liquid chromatography/tandem mass spectrometry. J. Chromatogr. B 949-950, 70–78. 10.1016/j.jchromb.2013.12.039 PMC4188440 24480327

[B10] Cao P. Li Y. Tang Y. Ding C. Hunter D. J. (2020). Pharmacotherapy for knee osteoarthritis: current and emerging therapies. Expert Opin. Pharmacother. 21 (7), 797–809. 10.1080/14656566.2020.1732924 32100600

[B11] Carlson A. K. Rawle R. A. Wallace C. W. Brooks E. G. Adams E. Greenwood M. C. (2019). Characterization of synovial fluid metabolomic phenotypes of cartilage morphological changes associated with osteoarthritis. Osteoarthritis Cartilage 27 (8), 1174–1184. 10.1016/j.joca.2019.04.007 31028882 PMC6646055

[B12] Cheleschi S. Cantarini L. Pascarelli N. A. Collodel G. Lucherini O. M. Galeazzi M. (2015). Possible chondroprotective effect of canakinumab: an *in vitro* study on human osteoarthritic chondrocytes. Cytokine 71 (2), 165–172. 10.1016/j.cyto.2014.10.023 25461395

[B13] Chen D. Shen J. Zhao W. Wang T. Han L. Hamilton J. L. (2017). Osteoarthritis: toward a comprehensive understanding of pathological mechanism. Bone Res. 5, 16044. 10.1038/boneres.2016.44 28149655 PMC5240031

[B14] Chen J. Li J. Li R. Wang H. Yang J. Xu J. (2017). Efficacy and safety of tanezumab on osteoarthritis knee and hip pains: a meta-analysis of randomized controlled trials. Pain Med. 18 (2), pnw262–385. 10.1093/pm/pnw262 28034979

[B15] Cherian J. J. Parvizi J. Bramlet D. Lee K. H. Romness D. W. Mont M. A. (2015). Preliminary results of a phase II randomized study to determine the efficacy and safety of genetically engineered allogeneic human chondrocytes expressing TGF-β1 in patients with grade 3 chronic degenerative joint disease of the knee. Osteoarthritis Cartilage 23 (12), 2109–2118. 10.1016/j.joca.2015.06.019 26188189

[B16] Chevalier X. Eymard F. (2019). Anti-IL-1 for the treatment of OA: dead or alive?. Nat. Rev. Rheumatol. 15 (4), 191–192. 10.1038/s41584-019-0185-y 30770908

[B17] Chevalier X. Goupille P. Beaulieu A. D. Burch F. X. Bensen W. G. Conrozier T. (2009). Intraarticular injection of anakinra in osteoarthritis of the knee: a multicenter, randomized, double-blind, placebo-controlled study. Arthritis Rheum. 61 (3), 344–352. 10.1002/art.24096 19248129

[B18] Chevalier X. Ravaud P. Maheu E. Baron G. Rialland A. Vergnaud P. (2015). Adalimumab in patients with hand osteoarthritis refractory to analgesics and NSAIDs: a randomised, multicentre, double-blind, placebo-controlled trial. Ann. Rheum. Dis. 74 (9), 1697–1705. 10.1136/annrheumdis-2014-205348 24817417

[B19] Childs B. G. Gluscevic M. Baker D. J. Laberge R.-M. Marquess D. Dananberg J. (2017). Senescent cells: an emerging target for diseases of ageing. Nat. Rev. Drug Discov. 16 (10), 718–735. 10.1038/nrd.2017.116 28729727 PMC5942225

[B20] Chiusaroli R. Visentini M. Galimberti C. Casseler C. Mennuni L. Covaceuszach S. (2013). Targeting of ADAMTS5's ancillary domain with the recombinant mAb CRB0017 ameliorates disease progression in a spontaneous murine model of osteoarthritis. Osteoarthritis and Cartilage 21 (11), 1807–1810. 10.1016/j.joca.2013.08.015 23954517

[B21] Cohen S. B. Proudman S. Kivitz A. J. Burch F. X. Donohue J. P. Burstein D. (2011). A randomized, double-blind study of AMG 108 (a fully human monoclonal antibody to IL-1R1) in patients with osteoarthritis of the knee. Arthritis Res. Ther. 13 (4), R125. 10.1186/ar3430 21801403 PMC3239365

[B22] Conaghan P. G. Bowes M. A. Kingsbury S. R. Brett A. Guillard G. Rizoska B. (2020). Disease-modifying effects of a novel cathepsin K inhibitor in osteoarthritis. Ann. Intern. Med. 172 (2), 86–95. 10.7326/M19-0675 31887743

[B23] Conaghan P. G. Cohen S. B. Berenbaum F. Lufkin J. Johnson J. R. Bodick N. (2018a). Brief report: a phase IIb trial of a novel extended-release microsphere formulation of triamcinolone acetonide for intraarticular injection in knee osteoarthritis. Arthritis Rheumatol. 70 (2), 204–211. 10.1002/art.40364 29088579 PMC5814922

[B24] Conaghan P. G. Hunter D. J. Cohen S. B. Kraus V. B. Berenbaum F. Lieberman J. R. (2018b). Effects of a single intra-articular injection of a microsphere formulation of triamcinolone acetonide on knee osteoarthritis pain. J. Bone Jt. Surg. 100 (8), 666–677. 10.2106/JBJS.17.00154 PMC5916484 29664853

[B25] Coryell P. R. Diekman B. O. Loeser R. F. (2020). Mechanisms and therapeutic implications of cellular senescence in osteoarthritis. Nat. Rev. Rheumatol. 17, 47. 10.1038/s41584-020-00533-7 33208917 PMC8035495

[B26] Coulombe J. Faure H. Robin B. Ruat M. (2004). *In vitro* effects of strontium ranelate on the extracellular calcium-sensing receptor. Biochem. Biophysical Res. Commun. 323 (4), 1184–1190. 10.1016/j.bbrc.2004.08.209 15451421

[B27] Crane J. L. Xian L. Cao X. (2016). Role of TGF-β signaling in coupling bone remodeling. Methods Mol. Biol. 1344, 287–300. 10.1007/978-1-4939-2966-5_18 26520132

[B28] da Costa B. R. Reichenbach S. Keller N. Nartey L. Wandel S. Jüni P. (2017). Effectiveness of non-steroidal anti-inflammatory drugs for the treatment of pain in knee and hip osteoarthritis: a network meta-analysis. Lancet 390 (10090), e21–e33. 10.1016/S0140-6736(17)31744-0 28699595

[B29] Dakin P. DiMartino S. J. Gao H. Maloney J. Kivitz A. J. Schnitzer T. J. (2019). The efficacy, tolerability, and joint safety of Fasinumab in osteoarthritis pain: a phase IIb/III double‐blind, placebo‐controlled, randomized clinical trial. Arthritis Rheumatol. 71 (11), 1824–1834. 10.1002/art.41012 31207169 PMC6900077

[B30] Dejica V. M. Mort J. S. Laverty S. Percival M. D. Antoniou J. Zukor D. J. (2008). Cleavage of type II collagen by cathepsin K in human osteoarthritic cartilage. Am. J. Pathol. 173 (1), 161–169. 10.2353/ajpath.2008.070494 18511517 PMC2438294

[B31] Denk F. Bennett D. L. McMahon S. B. (2017). Nerve growth factor and pain mechanisms. Annu. Rev. Neurosci. 40, 307–325. 10.1146/annurev-neuro-072116-031121 28441116

[B32] Deshmukh V. Hu H. Barroga C. Bossard C. Kc S. Dellamary L. (2018). A small-molecule inhibitor of the Wnt pathway (SM04690) as a potential disease modifying agent for the treatment of osteoarthritis of the knee. Osteoarthritis Cartilage 26 (1), 18–27. 10.1016/j.joca.2017.08.015 28888902

[B33] Deshmukh V. O'Green A. L. Bossard C. Seo T. Lamangan L. Ibanez M. (2019). Modulation of the Wnt pathway through inhibition of CLK2 and DYRK1A by lorecivivint as a novel, potentially disease-modifying approach for knee osteoarthritis treatment. Osteoarthritis and Cartilage 27 (9), 1347–1360. 10.1016/j.joca.2019.05.006 31132406

[B34] Deveza L. A. Nelson A. E. Loeser R. F. (2019). Phenotypes of osteoarthritis: current state and future implications. Clin. Exp. Rheumatol. 37 (Suppl. 120), 64–72. 31621574 PMC6936212

[B35] Ding C. Cicuttini F. Parameswaran V. Burgess J. Quinn S. Jones G. (2009). Serum levels of vitamin D, sunlight exposure, and knee cartilage loss in older adults: the Tasmanian older adult cohort study. Arthritis Rheum. 60 (5), 1381–1389. 10.1002/art.24486 19404958

[B36] Duong L. T. Leung A. T. Langdahl B. (2016). Cathepsin K inhibition: a new mechanism for the treatment of osteoporosis. Calcif Tissue Int. 98 (4), 381–397. 10.1007/s00223-015-0051-0 26335104

[B37] Eckstein F. Le Graverand M.-P. H. (2015). Plain radiography or magnetic resonance imaging (MRI): which is better in assessing outcome in clinical trials of disease-modifying osteoarthritis drugs? Summary of a debate held at the World Congress of Osteoarthritis 2014. Semin. Arthritis Rheum. 45 (3), 251–256. 10.1016/j.semarthrit.2015.06.001 26142321

[B38] Eckstein F. Wirth W. Guermazi A. Maschek S. Aydemir A. (2015). Brief report: intraarticular sprifermin not only increases cartilage thickness, but also reduces cartilage loss: location‐independent post hoc analysis using magnetic resonance imaging. Arthritis Rheumatol. 67 (11), 2916–2922. 10.1002/art.39265 26138203 PMC5061102

[B39] Eckstein F. Kraines J. L. Aydemir A. Wirth W. Maschek S. Hochberg M. C. (2020). Intra-articular sprifermin reduces cartilage loss in addition to increasing cartilage gain independent of location in the femorotibial joint: post-hoc analysis of a randomised, placebo-controlled phase II clinical trial. Ann. Rheum. Dis. 79 (4), 525–528. 10.1136/annrheumdis-2019-216453 32098758 PMC7147175

[B40] Felson D. T. (2006). Osteoarthritis of the knee. N. Engl. J. Med. 354 (8), 841–848. 10.1056/NEJMcp051726 16495396

[B41] Fioravanti A. Fabbroni M. Cerase A. Galeazzi M. (2009). Treatment of erosive osteoarthritis of the hands by intra-articular infliximab injections: a pilot study. Rheumatol. Int. 29 (8), 961–965. 10.1007/s00296-009-0872-0 19198842

[B42] Fleischmann R. M. Bliddal H. Blanco F. J. Schnitzer T. J. Peterfy C. Chen S. (2019). A phase II trial of lutikizumab, an anti-interleukin‐1α/β dual variable domain immunoglobulin, in knee osteoarthritis patients with synovitis. Arthritis Rheumatol. 71 (7), 1056–1069. 10.1002/art.40840 30653843

[B43] Fuggle N. Curtis E. Shaw S. Spooner L. Bruyère O. Ntani G. (2019). Safety of opioids in osteoarthritis: outcomes of a systematic review and meta-analysis. Drugs Aging 36 (Suppl. 1), 129–143. 10.1007/s40266-019-00666-9 31073926 PMC6509215

[B44] Ghouri A. Conaghan P. G. (2019). Treating osteoarthritis pain: recent approaches using pharmacological therapies. Clin. Exp. Rheumatol. 37 Suppl 120 (5), 124–129. 31621576

[B45] Gigout A. Guehring H. Froemel D. Meurer A. Ladel C. Reker D. (2017). Sprifermin (rhFGF18) enables proliferation of chondrocytes producing a hyaline cartilage matrix. Osteoarthritis Cartilage 25 (11), 1858–1867. 10.1016/j.joca.2017.08.004 28823647

[B46] Güler-Yüksel M. Allaart C. F. Watt I. Goekoop-Ruiterman Y. P. M. de Vries-Bouwstra J. K. van Schaardenburg D. (2010). Treatment with TNF-α inhibitor infliximab might reduce hand osteoarthritis in patients with rheumatoid arthritis. Osteoarthritis Cartilage 18 (10), 1256–1262. 10.1016/j.joca.2010.07.011 20691795

[B47] Ha C.-W. Noh M. J. Choi K. B. Lee K. H. (2012). Initial phase I safety of retrovirally transduced human chondrocytes expressing transforming growth factor-beta-1 in degenerative arthritis patients. Cytotherapy 14 (2), 247–256. 10.3109/14653249.2011.629645 22242865 PMC3793276

[B48] Ha C. W. Cho J. J. Elmallah R. K. Cherian J. J. Kim T. W. Lee M. C. (2015). A multicenter, single-blind, phase IIa clinical trial to evaluate the efficacy and safety of a cell-mediated gene therapy in degenerative knee arthritis patients. Hum. Gene Ther. Clin. Dev. 26 (2), 125–130. 10.1089/humc.2014.145 25760423

[B49] Han W. Fan S. Bai X. Ding C. (2017). Strontium ranelate, a promising disease modifying osteoarthritis drug. Expert Opin. Investig. Drugs 26 (3), 375–380. 10.1080/13543784.2017.1283403 28092725

[B50] Hochberg M. C. Guermazi A. Guehring H. Aydemir A. Wax S. Fleuranceau-Morel P. (2019). Effect of intra-articular sprifermin vs placebo on femorotibial joint cartilage thickness in patients with osteoarthritis. JAMA 322 (14), 1360–1370. 10.1001/jama.2019.14735 31593273 PMC6784851

[B51] Hochberg M. C. (2015). Serious joint-related adverse events in randomized controlled trials of anti-nerve growth factor monoclonal antibodies. Osteoarthritis Cartilage 23 (Suppl. 1), S18–S21. 10.1016/j.joca.2014.10.005 25527216

[B52] Hochberg M. C. Tive L. A. Abramson S. B. Vignon E. Verburg K. M. West C. R. (2016). When is osteonecrosis not osteonecrosis?: adjudication of reported serious adverse joint events in the tanezumab clinical development program. Arthritis Rheumatol. 68 (2), 382–391. 10.1002/art.39492 26554876

[B53] Hunter D. J. Bierma-Zeinstra S. (2019). Osteoarthritis. Lancet 393 (10182), 1745–1759. 10.1016/S0140-6736(19)30417-9 31034380

[B54] Jayabalan P. Schnitzer T. J. (2017). Tanezumab in the treatment of chronic musculoskeletal conditions. Expert Opin. Biol. Ther. 17 (2), 245–254. 10.1080/14712598.2017.1271873 27936977 PMC7462362

[B55] Jeon O. H. Kim C. Laberge R.-M. Demaria M. Rathod S. Vasserot A. P. (2017). Local clearance of senescent cells attenuates the development of post-traumatic osteoarthritis and creates a pro-regenerative environment. Nat. Med. 23 (6), 775–781. 10.1038/nm.4324 28436958 PMC5785239

[B56] Jeon O. H. David N. Campisi J. Elisseeff J. H. (2018). Senescent cells and osteoarthritis: a painful connection. J. Clin. Invest. 128 (4), 1229–1237. 10.1172/JCI95147 29608139 PMC5873863

[B57] Jin X. Jones G. Cicuttini F. Wluka A. Zhu Z. Han W. (2016). Effect of vitamin D supplementation on tibial cartilage volume and knee pain among patients with symptomatic knee osteoarthritis. JAMA 315 (10), 1005–1013. 10.1001/jama.2016.1961 26954409

[B58] Karsdal M. A. Bay-Jensen A. C. Lories R. J. Abramson S. Spector T. Pastoureau P. (2014). The coupling of bone and cartilage turnover in osteoarthritis: opportunities for bone antiresorptives and anabolics as potential treatments?. Ann. Rheum. Dis. 73 (2), 336–348. 10.1136/annrheumdis-2013-204111 24285494

[B59] Karsdal M. A. Byrjalsen I. Alexandersen P. Bihlet A. Andersen J. R. Riis B. J. (2015). Treatment of symptomatic knee osteoarthritis with oral salmon calcitonin: results from two phase 3 trials. Osteoarthritis Cartilage 23 (4), 532–543. 10.1016/j.joca.2014.12.019 25582279

[B60] Karsdal M. A. Michaelis M. Ladel C. Siebuhr A. S. Bihlet A. R. Andersen J. R. (2016). Disease-modifying treatments for osteoarthritis (DMOADs) of the knee and hip: lessons learned from failures and opportunities for the future. Osteoarthritis Cartilage 24 (12), 2013–2021. 10.1016/j.joca.2016.07.017 27492463

[B61] Kim M. K. Ha C. W. In Y. In S. D. Choi E. S. Ha J. K. (2018). A multicenter, double-blind, phase III clinical trial to evaluate the efficacy and safety of a cell and gene therapy in knee osteoarthritis patients. Hum. Gene Ther. Clin. Dev. 29 (1), 48–59. 10.1089/humc.2017.249 29641281

[B62] Kim Y. Levin G. Nikolov N. P. Abugov R. Rothwell R. (2020). Concept endpoints informing design considerations for confirmatory clinical trials in osteoarthritis. Arthritis Care Res. 10.1002/acr.24549 33345469

[B63] Kingsbury S. R. Tharmanathan P. Arden N. K. Batley M. Birrell F. Cocks K. (2015). Pain reduction with oral methotrexate in knee osteoarthritis, a pragmatic phase iii trial of treatment effectiveness (PROMOTE): study protocol for a randomized controlled trial. Trials 16, 77. 10.1186/s13063-015-0602-8 25872649 PMC4351849

[B64] Kingsbury S. R. Tharmanathan P. Keding A. Ronaldson S. J. Grainger A. Wakefield R. J. (2018). Hydroxychloroquine effectiveness in reducing symptoms of hand osteoarthritis. Ann. Intern. Med. 168 (6), 385–395. 10.7326/M17-1430 29459986

[B65] Kloppenburg M. Ramonda R. Bobacz K. Kwok W.-Y. Elewaut D. Huizinga T. W. J. (2018). Etanercept in patients with inflammatory hand osteoarthritis (EHOA): a multicentre, randomised, double-blind, placebo-controlled trial. Ann. Rheum. Dis. 77 (12), 1757–1764. 10.1136/annrheumdis-2018-213202 30282670

[B66] Kloppenburg M. Peterfy C. Haugen I. K. Kroon F. Chen S. Wang L. (2019). Phase IIa, placebo-controlled, randomised study of lutikizumab, an anti-interleukin-1α and anti-interleukin-1β dual variable domain immunoglobulin, in patients with erosive hand osteoarthritis. Ann. Rheum. Dis. 78 (3), 413–420. 10.1136/annrheumdis-2018-213336 30552176 PMC6390132

[B67] Kraus V. B. Conaghan P. G. Aazami H. A. Mehra P. Kivitz A. J. Lufkin J. (2018). Synovial and systemic pharmacokinetics (PK) of triamcinolone acetonide (TA) following intra-articular (IA) injection of an extended-release microsphere-based formulation (FX006) or standard crystalline suspension in patients with knee osteoarthritis (OA). Osteoarthritis and Cartilage 26 (1), 34–42. 10.1016/j.joca.2017.10.003 29024802

[B68] Kroon F. Bay-Jensen A. Wittoek R. Verbruggen G. Smolen J. Kloppenburg M. (2020). Etanercept therapy leads to reductions in matrix metalloproteinase-3 in patients with erosive hand osteoarthritis. Scand. J. Rheumatol. 49 (2), 167–168. 10.1080/03009742.2019.1657493 31566063

[B69] Kuttapitiya A. Assi L. Laing K. Hing C. Mitchell P. Whitley G. (2017). Microarray analysis of bone marrow lesions in osteoarthritis demonstrates upregulation of genes implicated in osteochondral turnover, neurogenesis and inflammation. Ann. Rheum. Dis. 76 (10), 1764–1773. 10.1136/annrheumdis-2017-211396 28705915 PMC5629942

[B70] Lacy S. E. Wu C. Ambrosi D. J. Hsieh C.-M. Bose S. Miller R. (2015). Generation and characterization of ABT-981, a dual variable domain immunoglobulin (DVD-IgTM) molecule that specifically and potently neutralizes both IL-1α and IL-1β. MAbs 7 (3), 605–619. 10.1080/19420862.2015.1026501 25764208 PMC4622731

[B71] Lane N. E. Corr M. (2017). Anti-NGF treatments for pain - two steps forward, one step back?. Nat. Rev. Rheumatol. 13 (2), 76–78. 10.1038/nrrheum.2016.224 28119540

[B72] Larkin J. Lohr T. A. Elefante L. Shearin J. Matico R. Su J.-L. (2015). Translational development of an ADAMTS-5 antibody for osteoarthritis disease modification. Osteoarthritis and Cartilage 23 (8), 1254–1266. 10.1016/j.joca.2015.02.778 25800415 PMC4516626

[B73] Latourte A. Kloppenburg M. Richette P. (2020). Emerging pharmaceutical therapies for osteoarthritis. Nat. Rev. Rheumatol. 16 (12), 673–688. 10.1038/s41584-020-00518-6 33122845

[B74] Lee W. Ruijgrok L. Boxma-de Klerk B. Kok M. R. Kloppenburg M. Gerards A. (2018). Efficacy of hydroxychloroquine in hand osteoarthritis: a randomized, double-blind, placebo-controlled trial. Arthritis Care Res. 70 (9), 1320–1325. 10.1002/acr.23471 29125901

[B75] LeGrand A. Fermor B. Fink C. Pisetsky D. S. Weinberg J. B. Vail T. P. (2001). Interleukin-1, tumor necrosis factor ?, and interleukin-17 synergistically up-regulate nitric oxide and prostaglandin E2 production in explants of human osteoarthritic knee menisci **.** Arthritis Rheum. 44 (9), 2078–2083. 10.1002/1529-0131(200109)44:9<2078::aid-art358>3.0.co;2-j 11592370

[B76] Leopoldino A. O. Machado G. C. Ferreira P. H. Pinheiro M. B. Day R. McLachlan A. J. (2019). Paracetamol versus placebo for knee and hip osteoarthritis. Cochrane Database Syst. Rev. 2, D13273. 10.1002/14651858.CD013273 PMC6388567 30801133

[B77] Li H. Ding X. Terkeltaub R. Lin H. Zhang Y. Zhou B. (2020). Exploration of metformin as novel therapy for osteoarthritis: preventing cartilage degeneration and reducing pain behavior. Arthritis Res. Ther. 22 (1). 10.1186/s13075-020-2129-y PMC7036179 32087740

[B78] Li J. Zhang B. Liu W.-X. Lu K. Pan H. Wang T. (2020). Metformin limits osteoarthritis development and progression through activation of AMPK signalling. Ann. Rheum. Dis. 79 (5), 635–645. 10.1136/annrheumdis-2019-216713 32156705 PMC7213329

[B79] Lindström E. Rizoska B. Henderson I. Terelius Y. Jerling M. Edenius C. (2018a). Nonclinical and clinical pharmacological characterization of the potent and selective cathepsin K inhibitor MIV-711. J. Transl Med. 16 (1), 125. 10.1186/s12967-018-1497-4 29743078 PMC5944028

[B80] Lindström E. Rizoska B. Tunblad K. Edenius C. Bendele A. M. Maul D. (2018b). The selective cathepsin K inhibitor MIV-711 attenuates joint pathology in experimental animal models of osteoarthritis. J. Transl Med. 16 (1), 56. 10.1186/s12967-018-1425-7 29523155 PMC5845353

[B81] Liu W. Feng M. Jayasuriya C. T. Peng H. Zhang L. Guan Y. (2020). Human osteoarthritis cartilage‐derived stromal cells activate joint degeneration through TGF‐beta lateral signaling. FASEB j. 34 (12), 16552–16566. 10.1096/fj.202001448R 33118211 PMC8219090

[B82] Loeser R. F. Goldring S. R. Scanzello C. R. Goldring M. B. (2012). Osteoarthritis: a disease of the joint as an organ. Arthritis Rheum. 64 (6), 1697–1707. 10.1002/art.34453 22392533 PMC3366018

[B83] Lohmander L. S. Hellot S. Dreher D. Krantz E. F. W. Kruger D. S. Guermazi A. (2014). Intraarticular sprifermin (recombinant human fibroblast growth factor 18) in knee osteoarthritis: a randomized, double-blind, placebo-controlled trial. Arthritis Rheumatol. 66 (7), 1820–1831. 10.1002/art.38614 24740822

[B84] Lories R. J. Corr M. Lane N. E. (2013). To Wnt or not to Wnt: the bone and joint health dilemma. Nat. Rev. Rheumatol. 9 (6), 328–339. 10.1038/nrrheum.2013.25 23459013 PMC4830431

[B85] Lu C.-H. Chung C.-H. Lee C.-H. Hsieh C.-H. Hung Y.-J. Lin F.-H. (2018). Combination COX-2 inhibitor and metformin attenuate rate of joint replacement in osteoarthritis with diabetes: a nationwide, retrospective, matched-cohort study in Taiwan. PLoS One 13 (1), e0191242. 10.1371/journal.pone.0191242 29385156 PMC5791980

[B86] Ma C. H. Lv Q. Yu Y. X. Zhang Y. Kong D. Niu K. R. (2015). Protective effects of tumor necrosis factor-α blockade by adalimumab on articular cartilage and subchondral bone in a rat model of osteoarthritis. Braz. J. Med. Biol. Res. 48 (10), 863–870. 10.1590/1414-431X20154407 26445328 PMC4617111

[B87] Macica C. Liang G. Nasiri A. Broadus A. E. (2011). Genetic evidence of the regulatory role of parathyroid hormone-related protein in articular chondrocyte maintenance in an experimental mouse model. Arthritis Rheum. 63 (11), 3333–3343. 10.1002/art.30515 21702022 PMC3197958

[B88] Malfait A.-M. Schnitzer T. J. (2013). Towards a mechanism-based approach to pain management in osteoarthritis. Nat. Rev. Rheumatol. 9 (11), 654–664. 10.1038/nrrheum.2013.138 24045707 PMC4151882

[B89] Malfait A. M. Tortorella M. D. (2019). The "elusive DMOAD": aggrecanase inhibition from laboratory to clinic. Clin. Exp. Rheumatol. 37 Suppl 120 (5), 130–134. 31621572

[B90] McAlindon T. E. Bannuru R. R. (2010). OARSI recommendations for the management of hip and knee osteoarthritis: the semantics of differences and changes. Osteoarthritis Cartilage 18 (4), 473–475. 10.1016/j.joca.2010.02.011 20171299

[B91] McAlindon T. LaValley M. Schneider E. Nuite M. Lee J. Y. Price L. L. (2013). Effect of vitamin D supplementation on progression of knee pain and cartilage volume loss in patients with symptomatic osteoarthritis. JAMA 309 (2), 155–162. 10.1001/jama.2012.164487 23299607 PMC3984919

[B92] McAlindon T. E. LaValley M. P. Harvey W. F. Price L. L. Driban J. B. Zhang M. (2017). Effect of intra-articular triamcinolone vs saline on knee cartilage volume and pain in patients with knee osteoarthritis. JAMA 317 (19), 1967–1975. 10.1001/jama.2017.5283 28510679 PMC5815012

[B93] Miller R. E. Miller R. J. Malfait A.-M. (2014). Osteoarthritis joint pain: the cytokine connection. Cytokine 70 (2), 185–193. 10.1016/j.cyto.2014.06.019 25066335 PMC4254338

[B94] Miller R. E. Tran P. B. Ishihara S. Larkin J. Malfait A. M. (2016). Therapeutic effects of an anti-ADAMTS-5 antibody on joint damage and mechanical allodynia in a murine model of osteoarthritis. Osteoarthritis Cartilage 24 (2), 299–306. 10.1016/j.joca.2015.09.005 26410555 PMC4743933

[B95] Miller R. E. Block J. A. Malfait A.-M. (2017). Nerve growth factor blockade for the management of osteoarthritis pain: what can we learn from clinical trials and preclinical models? Curr. Opin. Rheumatol. 29 (1), 110–118. 10.1097/BOR.0000000000000354 27672741 PMC5436144

[B96] Miller R. E. Block J. A. Malfait A.-M. (2018). What is new in pain modification in osteoarthritis?. Rheumatology (Oxford) 57 (Suppl. l_4), iv99–iv107. 10.1093/rheumatology/kex522 29361112 PMC5905627

[B97] Millerand M. Berenbaum F. Jacques C. (2019). Danger signals and inflammaging in osteoarthritis. Clin. Exp. Rheumatol. 37 Suppl 120 (Suppl. 1205), 48–56. 31621566

[B98] Moore E. E. Bendele A. M. Thompson D. L. Littau A. Waggie K. S. Reardon B. (2005). Fibroblast growth factor-18 stimulates chondrogenesis and cartilage repair in a rat model of injury-induced osteoarthritis. Osteoarthritis Cartilage 13 (7), 623–631. 10.1016/j.joca.2005.03.003 15896984

[B99] Nelson A. E. Fang F. Arbeeva L. Cleveland R. J. Schwartz T. A. Callahan L. F. (2019). A machine learning approach to knee osteoarthritis phenotyping: data from the FNIH Biomarkers Consortium. Osteoarthritis Cartilage 27 (7), 994–1001. 10.1016/j.joca.2018.12.027 31002938 PMC6579689

[B100] Nwosu L. N. Gowler P. R. W. Burston J. J. Rizoska B. Tunblad K. Lindström E. (2018). Analgesic effects of the cathepsin K inhibitor L-006235 in the monosodium iodoacetate model of osteoarthritis pain. Pain Rep. 3 (6), e685. 10.1097/PR9.0000000000000685 30706033 PMC6344135

[B101] Ohtori S. Orita S. Yamauchi K. Eguchi Y. Ochiai N. Kishida S. (2015). Efficacy of direct injection of Etanercept into knee joints for pain in moderate and severe knee osteoarthritis. Yonsei Med. J. 56 (5), 1379–1383. 10.3349/ymj.2015.56.5.1379 26256983 PMC4541670

[B102] Onakpoya I. J. Spencer E. A. Perera R. Heneghan C. J. (2017). Effectiveness of curcuminoids in the treatment of knee osteoarthritis: a systematic review and meta-analysis of randomized clinical trials. Int. J. Rheum. Dis. 20 (4), 420–433. 10.1111/1756-185X.13069 28470851

[B103] Onuora S. (2014). Sprifermin shows cartilage-protective effects in knee OA. Nat. Rev. Rheumatol. 10 (6), 322. 10.1038/nrrheum.2014.68 24798571

[B104] Oo W. M. Hunter D. J. (2019). Disease modification in osteoarthritis: are we there yet?. Clin. Exp. Rheumatol. 37 Suppl 120 (5), 135–140. 31621568

[B105] Oo W. M. Yu S. P.-C. Daniel M. S. Hunter D. J. (2018). Disease-modifying drugs in osteoarthritis: current understanding and future therapeutics. Expert Opin. Emerging Drugs 23 (4), 331–347. 10.1080/14728214.2018.1547706 30415584

[B106] Orita S. Koshi T. Mitsuka T. Miyagi M. Inoue G. Arai G. (2011). Associations between proinflammatory cytokines in the synovial fluid and radiographic grading and pain-related scores in 47 consecutive patients with osteoarthritis of the knee. BMC Musculoskelet. Disord. 12, 144. 10.1186/1471-2474-12-144 21714933 PMC3144455

[B107] Peat G. Thomas M. J. (2020). Osteoarthritis year in review 2020: epidemiology & therapy. Osteoarthritis Cartilage 29, 180–189. 10.1016/j.joca.2020.10.007 33242603

[B108] Pelletier J. P. Roubille C. Raynauld J. P. Abram F. Dorais M. Delorme P. (2015). Disease-modifying effect of strontium ranelate in a subset of patients from the Phase III knee osteoarthritis study SEKOIA using quantitative MRI: reduction in bone marrow lesions protects against cartilage loss. Ann. Rheum. Dis. 74 (2), 422–429. 10.1136/annrheumdis-2013-203989 24297379

[B109] Piecha D. Weik J. Kheil H. Becher G. Timmermann A. Jaworski A. (2010). Novel selective MMP-13 inhibitors reduce collagen degradation in bovine articular and human osteoarthritis cartilage explants. Inflamm. Res. 59 (5), 379–389. 10.1007/s00011-009-0112-9 19902332

[B110] Pincus T. Castrejon I. Yazici Y. Gibson K. A. Bergman M. J. Block J. A. (2019). Osteoarthritis is as severe as rheumatoid arthritis: evidence over 40 years according to the same measure in each disease. Clin. Exp. Rheumatol. 37 Suppl 120 (5), 7–17. 31621569

[B111] Reginster J.-Y. Badurski J. Bellamy N. Bensen W. Chapurlat R. Chevalier X. (2013). Efficacy and safety of strontium ranelate in the treatment of knee osteoarthritis: results of a double-blind, randomised placebo-controlled trial. Ann. Rheum. Dis. 72 (2), 179–186. 10.1136/annrheumdis-2012-202231 23117245 PMC3599139

[B112] Reginster J.-Y. Reiter-Niesert S. Bruyère O. Berenbaum F. Brandi M.-L. Branco J. (2015). Recommendations for an update of the 2010 European regulatory guideline on clinical investigation of medicinal products used in the treatment of osteoarthritis and reflections about related clinically relevant outcomes: expert consensus statement. Osteoarthritis Cartilage 23 (12), 2086–2093. 10.1016/j.joca.2015.07.001 26187570

[B113] Reker D. Kjelgaard-Petersen C. F. Siebuhr A. S. Michaelis M. Gigout A. Karsdal M. A. (2017). Sprifermin (rhFGF18) modulates extracellular matrix turnover in cartilage explants *ex vivo* . J. Transl Med. 15 (1), 250. 10.1186/s12967-017-1356-8 29233174 PMC5727954

[B114] Roemer F. W. Aydemir A. Lohmander S. Crema M. D. Marra M. D. Muurahainen N. (2016). Structural effects of sprifermin in knee osteoarthritis: a post-hoc analysis on cartilage and non-cartilaginous tissue alterations in a randomized controlled trial. BMC Musculoskelet. Disord. 17, 267. 10.1186/s12891-016-1128-2 27393009 PMC4938999

[B115] Roman-Blas J. A. Mendoza-Torres L. A. Largo R. Herrero-Beaumont G. (2020). Setting up distinctive outcome measures for each osteoarthritis phenotype. Ther. Adv. Musculoskelet. 12, 1759720X20937966. 10.1177/1759720X20937966 PMC7491224 32973934

[B116] Rünger T. M. Adami S. Benhamou C. L. Czerwiński E. Farrerons J. Kendler D. L. (2012). Morphea-like skin reactions in patients treated with the cathepsin K inhibitor balicatib. J. Am. Acad. Dermatol. 66 (3), e89–e96. 10.1016/j.jaad.2010.11.033 21571394

[B117] Salminen-Mankonen H. Morko J. Vuorio E. (2007). Role of cathepsin K in normal joints and in the development of arthritis. Curr. Drug Targets 8 (2), 315–323. 10.2174/138945007779940188 17305509

[B118] Sampson E. R. Hilton M. J. Tian Y. Chen D. Schwarz E. M. Mooney R. A. (2011). Teriparatide as a chondroregenerative therapy for injury-induced osteoarthritis. Sci. Transl. Med. 3 (101), 101ra93. 10.1126/scitranslmed.3002214 PMC3208271 21937758

[B119] Scanzello C. R. Loeser R. F. (2015). Editorial: inflammatory activity in symptomatic knee osteoarthritis: not all inflammation is local. Arthritis Rheumatol. 67 (11), 2797–2800. 10.1002/art.39304 26246460 PMC5093334

[B120] Schett G. Dayer J.-M. Manger B. (2016). Interleukin-1 function and role in rheumatic disease. Nat. Rev. Rheumatol. 12 (1), 14–24. 10.1038/nrrheum.2016.166 26656658

[B121] Schnitzer T. J. Easton R. Pang S. Levinson D. J. Pixton G. Viktrup L. (2019). Effect of tanezumab on joint pain, physical function, and patient global assessment of osteoarthritis among patients with osteoarthritis of the hip or knee. JAMA 322 (1), 37–48. 10.1001/jama.2019.8044 31265100 PMC6613301

[B122] Schnute M. E. O’Brien P. M. Nahra J. Morris M. Howard Roark W. Hanau C. E. (2010). Discovery of (pyridin-4-yl)-2H-tetrazole as a novel scaffold to identify highly selective matrix metalloproteinase-13 inhibitors for the treatment of osteoarthritis. Bioorg. Med. Chem. Lett. 20 (2), 576–580. 10.1016/j.bmcl.2009.11.081 20005097

[B123] Sennett M. L. Meloni G. R. Farran A. J. E. Guehring H. Mauck R. L. Dodge G. R. (2018). Sprifermin treatment enhances cartilage integration in an *in vitro* repair model. J. Orthop. Res. 36 (10), 2648–2656. 10.1002/jor.24048 29761549 PMC7241943

[B124] Shen L. Ji H. F. (2012). The pharmacology of curcumin: is it the degradation products?. Trends Mol. Med. 18 (3), 138–144. 10.1016/j.molmed.2012.01.004 22386732

[B125] Siebuhr A. S. Werkmann D. Bay-Jensen A.-C. Thudium C. S. Karsdal M. A. Serruys B. (2020). The anti-ADAMTS-5 Nanobody M6495 protects cartilage degradation ex vivo. Int. J. Mol. Sci. 21 (17), 5992. 10.3390/ijms21175992 PMC7503673 32825512

[B126] Sohn D. Sokolove J. Sharpe O. Erhart J. C. Chandra P. E. Lahey L. J. (2012). Plasma proteins present in osteoarthritic synovial fluid can stimulate cytokine production via Toll-like receptor 4. Arthritis Res. Ther. 14 (1), R7. 10.1186/ar3555 22225630 PMC3392793

[B127] Sota J. Vitale A. Vitale A. Insalaco A. Sfriso P. Lopalco G. (2018). Safety profile of the interleukin-1 inhibitors anakinra and canakinumab in real-life clinical practice: a nationwide multicenter retrospective observational study. Clin. Rheumatol. 37 (8), 2233–2240. 10.1007/s10067-018-4119-x 29770930

[B128] Tat S. K. Pelletier J.-P. Mineau F. Caron J. Martel-Pelletier J. (2011). Strontium ranelate inhibits key factors affecting bone remodeling in human osteoarthritic subchondral bone osteoblasts. Bone 49 (3), 559–567. 10.1016/j.bone.2011.06.005 21700005

[B129] Thysen S. Luyten F. P. Lories R. J. (2015). Loss of Frzb and Sfrp1 differentially affects joint homeostasis in instability-induced osteoarthritis. Osteoarthritis Cartilage 23 (2), 275–279. 10.1016/j.joca.2014.10.010 25450854

[B130] Tu L. Zheng S. Cicuttini F. Jin X. Han W. Zhu Z. (2020). Effects of vitamin D supplementation on disabling foot pain in patients with symptomatic knee osteoarthritis. Arthritis Care Res. 10.1002/acr.24371 32623812

[B131] Usami Y. Gunawardena A. T. Iwamoto M. Enomoto-Iwamoto M. (2016). Wnt signaling in cartilage development and diseases: lessons from animal studies. Lab. Invest. 96 (2), 186–196. 10.1038/labinvest.2015.142 26641070 PMC4838282

[B132] Vaysbrot E. E. Osani M. C. Musetti M.-C. McAlindon T. E. Bannuru R. R. (2018). Are bisphosphonates efficacious in knee osteoarthritis? A meta-analysis of randomized controlled trials. Osteoarthritis Cartilage 26 (2), 154–164. 10.1016/j.joca.2017.11.013 29222056

[B133] Verbruggen G. Wittoek R. Cruyssen B. V. Elewaut D. (2012). Tumour necrosis factor blockade for the treatment of erosive osteoarthritis of the interphalangeal finger joints: a double blind, randomised trial on structure modification. Ann. Rheum. Dis. 71 (6), 891–898. 10.1136/ard.2011.149849 22128078 PMC3371224

[B134] Vincent T. L. (2020). Peripheral pain mechanisms in osteoarthritis. Pain 161 (Suppl. 1), S138–S146. 10.1097/j.pain.0000000000001923 33090747 PMC7434216

[B135] Wang C. C. Wang C. T. Tsai K. L. Chou C. L. Chao J. K. Huang H. Y. (2021). Effect of ultrasound-detected synovitis on therapeutic efficacy of hyaluronic acid injection for symptomatic knee osteoarthritis. Rheumatology (Oxford). 10.1093/rheumatology/keab020 PMC8487310 33493323

[B136] Wang J. Ma J. Gu J.-H. Wang F.-Y. Shang X. S. Tao H. R. (2017). Regulation of type II collagen, matrix metalloproteinase-13 and cell proliferation by interleukin-1β is mediated by curcumin via inhibition of NF-κB signaling in rat chondrocytes. Mol. Med. Rep. 16 (2), 1837–1845. 10.3892/mmr.2017.6771 28627596 PMC5562050

[B137] Wang S. X. Abramson S. B. Attur M. Karsdal M. A. Preston R. A. Lozada C. J. (2017). Safety, tolerability, and pharmacodynamics of an anti-interleukin-1α/β dual variable domain immunoglobulin in patients with osteoarthritis of the knee: a randomized phase 1 study. Osteoarthritis Cartilage 25 (12), 1952–1961. 10.1016/j.joca.2017.09.007 28964890

[B138] Wang X. Cicuttini F. Jin X. Wluka A. E. Han W. Zhu Z. (2017). Knee effusion-synovitis volume measurement and effects of vitamin D supplementation in patients with knee osteoarthritis. Osteoarthritis Cartilage 25 (8), 1304–1312. 10.1016/j.joca.2017.02.804 28274889

[B139] Wang Y. Hussain S. M. Wluka A. E. Lim Y. Z. Abram F. Pelletier J.-P. (2019). Association between metformin use and disease progression in obese people with knee osteoarthritis: data from the Osteoarthritis Initiative-a prospective cohort study. Arthritis Res. Ther. 21 (1). 10.1186/s13075-019-1915-x PMC6534888 31126352

[B140] Wang Z. Jones G. Winzenberg T. Cai G. Laslett L. L. Aitken D. (2020). Effectiveness of curcuma longa extract for the treatment of symptoms and effusion-synovitis of knee osteoarthritis. Ann. Intern. Med. 173 (11), 861–869. 10.7326/M20-0990 32926799

[B141] Yang X. Chen L. Xu X. Li C. Huang C. Deng C. X. (2001). TGF-β/Smad3 signals repress chondrocyte hypertrophic differentiation and are required for maintaining articular cartilage. J. Cell Biol. 153 (1), 35–46. 10.1083/jcb.153.1.35 11285272 PMC2185521

[B142] Yazici Y. McAlindon T. E. Fleischmann R. Gibofsky A. Lane N. E. Kivitz A. J. (2017). A novel Wnt pathway inhibitor, SM04690, for the treatment of moderate to severe osteoarthritis of the knee: results of a 24-week, randomized, controlled, phase 1 study. Osteoarthritis Cartilage 25 (10), 1598–1606. 10.1016/j.joca.2017.07.006 28711582

[B143] Yu D. G. Ding H. F. Mao Y. Q. Liu M. Yu B. Zhao X. (2013). Strontium ranelate reduces cartilage degeneration and subchondral bone remodeling in rat osteoarthritis model. Acta Pharmacol. Sin 34 (3), 393–402. 10.1038/aps.2012.167 23334238 PMC4002491

[B144] Zhang R. K. Li G. W. Zeng C. Lin C. X. Huang L. S. Huang G. X. (2018). Mechanical stress contributes to osteoarthritis development through the activation of transforming growth factor beta 1 (TGF-β1). Bone Jt. Res. 7 (11), 587–594. 10.1302/2046-3758.711.BJR-2018-0057.R1 PMC6269596 30581556

[B145] Zhang W. Nuki G. Moskowitz R. W. Abramson S. Altman R. D. Arden N. K. (2010). OARSI recommendations for the management of hip and knee osteoarthritis. Osteoarthritis Cartilage 18 (4), 476–499. 10.1016/j.joca.2010.01.013 20170770

[B146] Zheng S. Jin X. Cicuttini F. Wang X. Zhu Z. Wluka A. (2017). Maintaining vitamin D sufficiency is associated with improved structural and symptomatic outcomes in knee osteoarthritis. Am. J. Med. 130 (10), 1211–1218. 10.1016/j.amjmed.2017.04.038 28549923

[B147] Zheng S. Tu L. Cicuttini F. Han W. Zhu Z. Antony B. (2019). Effect of vitamin D supplementation on depressive symptoms in patients with knee osteoarthritis. J. Am. Med. Directors Assoc. 20 (12), 1634–1640. 10.1016/j.jamda.2018.09.006 30401608

[B148] Zhu Z. Yu Q. Leng X. Han W. Li Z. Huang C. (2020). Can low-dose methotrexate reduce effusion-synovitis and symptoms in patients with mid- to late-stage knee osteoarthritis? Study protocol for a randomised, double-blind, and placebo-controlled trial. Trials 21 (1), 795. 10.1186/s13063-020-04687-3 32938470 PMC7493135

